# Comparative analysis of the sputum microbiota in different COPD clinical states

**DOI:** 10.1038/s41598-026-53780-1

**Published:** 2026-06-08

**Authors:** Lamis Galal, Mohamed A. Eltokhy, Heba M. Abostate, Maha Eid Omran, Sahar M. R. Radwan

**Affiliations:** 1 Microbiology and Immunology Department, Faculty of Pharmacy , Girls Al-Azhar University, Cairo, Egypt; 2 Department of Immunotherapeutics and Biotechnology, Center for Tumor Immunology and Targeted Cancer Therapy, Texas Tech University Health Sciences Center, Abilene, TX 79601 USA; 3 Microbiology & Immunology Department, Faculty of Pharmacy, Egyptian Russian University, Cairo, Egypt; 4 Microbiology & Immunology Department, Faculty of Pharmacy, Al-Azhar University, Cairo, Egypt; 5 Microbiology and Immunology department, Faculty of Pharmacy, Nile Valley University, Fayoum, 63518 Egypt

**Keywords:** COPD, Exacerbation microbiome, Proteobacteria, *Paracocus*, Diseases, Medical research, Microbiology

## Abstract

Chronic obstructive pulmonary disease (COPD) is a well-known respiratory illness. COPD patients oscillate between a stable state and an exacerbated state that leads to disease deterioration. Studies suggest that respiratory microbiome dysbiosis plays a vital role in COPD pathogenesis. However, the exact microbial composition among different clinical states of COPD is still elusive. To determine and compare the respiratory microbiota composition in different COPD clinical states, namely, the stable state (S-COPD) and the acute exacerbated state (AE-COPD). In this prospective study, 74 samples were collected from COPD patients. The sputum microbiota was analyzed via 16 S rRNA gene sequencing, and only 35 samples were included due to bad reads or not in accordance with inclusion criteria: S-COPD patients (*n* = 18), and AE-COPD patients (*n* = 17). Bioinformatics analysis was used to determine changes in the microbiota among the comparison groups. The most abundant phyla among all the samples were Proteobacteria, Fusobacteria, Firmicutes, and Actinobacteria, with *Paracoccus*, *Streptomyces Leptotrichia Fusobacterium* and *Ruminococcaceae* being the most prevalent genera. Dissimilarity in abundance across the studied COPD states was observed, with significantly greater abundance of Proteobacteria and Fusobacteria in S-COPD patients and greater abundance of *Firmicutes* in AE-COPD patients at the phylum level. At the genus level, *Paracoccus*, *Fusobacterium*,* Streptococcus*,* Haemophilus*, and *Moraxella* were significantly different between the two groups and were more prevalent in S-COPD, whereas *Cellulosilyticum*,* Streptomyces*,* Leptotrichia*,* Ruminococcaceae_UCG_014*, and *Atopobium* were more prevalent in exacerbated individuals. Alpha diversity revealed greater diversity in stable versus exacerbated patients, and a PCoA plot of Bray‒Curtis and weighted UniFrac distances revealed that stable patients were highly clustered, whereas exacerbated patients were more disseminated. At the genus level, LEfSe analysis revealed the dominance of *Cellulosilytic*,* Liptotrichia*, and *Streptomyces* in the AE-COPD group, whereas the S-COPD group microbiome was dominated by the genera *Paracoccus*, *Fusobacterium*, *Streptococcus Haemophilus*, and *Moraxella (p* < 0.05). The results of the present study suggest that COPD patients have unique microbial profiles that differ across different states, with increased abundances of Proteobacteria, chiefly *Paracoccus*. These findings need more research to clarify the definite role of microbiome dysbiosis in COPD pathogenesis.

**Supplementary Information:**

The online version contains supplementary material available at 10.1038/s41598-026-53780-1.

## Introduction

Chronic obstructive pulmonary disease (COPD) is characterized by chronic airway inflammation leading to impaired lung function and limited airflow^1^. COPD is the fifth leading cause of death worldwide, and by 2030, it is expected to be the fourth leading cause of death^2^; consequently, COPD causes a heavy socioeconomic burden and has become an active research area. COPD patients either have mild symptoms and are in a stable disease state or experience episodes of acute worsening symptoms called acute exacerbations^3^. Exacerbations are triggered by air pollutants, infections, or unknown causes^4^. The lung airways contain diverse microbiome compositions that affect the susceptibility or pathogenesis of respiratory diseases^5^. Although bacteria have been detected in sputum cultures from COPD patients during both stable and, more commonly, exacerbation states, the changes in bacterial ecology and its relationship with disease pathogenesis and exacerbation are yet unclear^6^.

Traditional culture techniques have many limitations, such as poor sensitivity and uncultivable bacteria. Moreover, in some cases, colonizing bacteria grow easily from cultured samples and interfere with pathogenic bacteria, causing exacerbations^7^. Recently, advanced diagnostic approaches and the development of culture-independent techniques such as 16 S rRNA gene sequencing have provided an opportunity for in-depth studies of the lung microbiome, as these methods can detect uncultivable bacteria and provide data about the microbial composition, diversity, richness and potential functional role of microbiome members^8^. Several noninvasive and invasive procedures are used for studying the lung microbiome; sputum is the most commonly used method because it is noninvasive and easy to access^9^. In this study, 16 S rRNA sequencing and metagenomics analysis were used to study the constitution of the sputum microbiome in COPD patients and compare different COPD states Stable (S-COPD) and exacerbated (AE-COPD).

## Methods and statistics

### Study design

This was a prospective multi-center observational cohort study. Patients were enrolled at the time of hospital visits, and samples were collected prior to initiation of any hospital medication. Patients were assigned into either S-COPD or AE-COPD group according to their condition. This study took place in Hospital A as well as Hospital B, Cairo, Egypt. The study took place from June 2019 to January 2020. Seventy-four patients were included in the study while only 35 patients were analyzed due to, all specimens from Hospital A were excluded, and bad reads from the remaining samples. Reporting of this study follows the STORMS (Strengthening The Organization and Reporting of Microbiome Studies) guidelines, and the corresponding checklist has been included as a supplementary document (A8).

#### Ethical statement

In this study, all the procedures performed were reviewed and approved by the Ethical Committee of Al-Azhar University, Egypt. This study was conducted according to the 1964 Declaration of Helsinki and its later amendments. All participants provided written informed consent.

#### Participant selection

The participants in the study, adult male patients with confirmed diagnosis of COPD, came to the hospital outpatient clinics for routine follow-up scheduled visits, providing regular treatments, having respiratory symptoms or complaining from other medical conditions. The participants underwent examinations and were classified based on their clinical presentation in accordance with GOLD criteria^10^. Stable COPD (S-COPD) included patients with no acute worsening of respiratory symptoms, evaluated in the outpatient setting for routine follow-up or other non-exacerbation-related reasons. Acute exacerbation COPD (AE-COPD) include patients with acute worsening of respiratory symptoms beyond normal day-to-day variation, requiring a change in therapy and/or hospital admission.

Sociodemographic and clinical information, including smoking history and the existence of coexisting diseases, respiratory symptoms, exacerbation frequency and therapies, were recorded. The exclusion criteria include patients who received antibiotic therapy (for at least three months without the use of antibiotics for any other reason), immunosuppressive drugs or microbial preparations such as probiotics or prebiotics.; female patients; smokers; patients with history or clinical diagnosis of acute or chronic respiratory diseases, including bronchiectasis, asthma, cystic fibrosis, diffuse bronchiolitis, pulmonary tuberculosis, pulmonary embolism, pulmonary edema, pneumonia based on radiographic findings or had a body temperature exceeding 38.0 °C at admission; had an active malignancy; sufferring from chronic, clinically significant cardiovascular, diabetic, hepatic, renal, or gastrointestinal disorders and had confirmed or suspected immunosuppression or immunodeficiency, whether primary or acquired, including HIV infection .

#### Clinical sample collection

In this investigation, induced sputum samples were collected^11^. A total of 35 samples were assigned to two clinical states: S-COPD (*n* = 17) and AE-COPD (*n* = 18). The samples were taken early on the first day. Aliquots (0.5 ml) of sputum samples were taken in sterile sample bottles and stored at − 80 °C for DNA extraction, and the remaining samples were subjected to routine culture.

#### DNA extraction

With some modifications, such as the addition of lysozyme and dithiothreitol solutions, genomic DNA was extracted from each sputum sample via the commercial QIAamp^®^ DNA Mini Kit (Qiagen, Germany) as directed by the manufacturer. The quality and quantity of the extracted DNA were evaluated via a NanoDrop system (NanoDrop Technology, USA). It was then visualized via 2% agarose gel electrophoresis and stored at − 80 °C for later analysis.

#### PCR amplification and 16 S sequencing

16 S rRNA gene amplification was applied. The Human Microbiome Project Consortium (HMP) ( http://www.hmpdacc.org/tools_protocols/tools_protocols.php ) was used for the construction of primers and barcodes. Using the extracted DNA as a template and universal 16 S rRNA primers, PCR was performed to amplify the hypervariable V3–V4 regions of the 16 S rRNA gene. Using Illumina adapters, forward-primer 27 F (5-GCC TAC GGG AGG CAG CAG T) and reverse-primer 1462R (5-GGACTACHVGGGTATCTAATCC) were altered in accordance with a previously published process^12^. The PCR conditions were set as follows: a 30-second denaturation stage at 95 °C, a 30-second annealing step at 60 °C, a 30-second extension step at 72 °C, and a final elongation step at 72 °C for 5 min. The initial hot-start incubation was conducted at 94 °C for 3 min. Ethidium bromide staining and 2% agarose gel electrophoresis were used to analyze the amplicons. Using the Illumina Nextera XT Index Kit (Illumina, CA, USA), the libraries were assembled by fastening Illumina adapters to the amplifiers. Agencourt AMPure XP beads were used to clean the PCR amplicons in accordance with the manufacturer’s instructions (Beckman Coulter, Inc., CA, USA). Purified amplicon libraries were examined via an Agilent Bioanalyzer 2100 with an Agilent DNA 1000 Kit (Agilent, Palo Alto, CA, USA) to guarantee the elimination of primers and any nonspecific amplicons^13^. Using paired-end Illumina MiSeq sequencing on an Illumina MiSeq instrument (Illumina Inc., San Diego, CA, USA), the 16 S rRNA was sequenced at the IGA Technology Services Company (Udine, Italy)^14^.

#### Sequence library analysis

Amplicon sequences were demultiplexed via MiSeq Reporter v2.3 (Illumina) as a first quality step. 16 S rRNA gene sequencing produced raw paired-end sequences in FASTQ format. As a second stage, sequence processing and quality filtering were performed via the “Quantitative Insights into Microbial Ecology” (QIIME2R version 0.99.21)^15^ pipeline to extract taxonomic information. The “join paired ends.py” argument was used to fuse overlapping paired-end 16 S rRNA gene sequences. Sequences with ambiguous reads (N), low-quality sequences end with mismatched forward or reverse primers, failed sequence reads, barcodes, and primers were eliminated for quality control. Sequences under 200 bp were also trimmed via the “QIIME script split_libraries.py calls” parameter (quality score < 25). Next, chimeras were detected in these clean sequences via the “identify_chimeric_seqs.py” option. Using an identity criterion of 97%^16^, sequences were grouped into Amplicon Sequence Variant (ASV) clusters and aligned via the SILVA alignment database (http://www.arb-silva.de/*).*^17^. The truncation length parameters of DADA2 were p-trunc-len-f 280 and p-trunc-len-r 220.

#### Statistical analysis

All graphical representations and statistical analyses were performed with https://www.microbiomeanalyst.ca. The Kruskal‒Wallis (KW) test and the nonparametric Mann‒Whitney test were used to identify species with significant differences between two or more groups, respectively. Diversity and differential-abundance analyses used unrarefied ASV table with normalization (R package). Four measures were used to assess bacterial α diversity: observed ASVs, the Chao1 richness measure, the Shannon index and the Simpson index diversity measure. To investigate the differences in the bacterial communities among COPD patients at various phases of species complexity, beta diversity analysis was utilized. On the basis of clinical variables and the relative abundance of taxa at the phylum, genus, and ASVs levels, PERMANOVA was used to determine the statistical significance of the groupings. The results are displayed via PCoA plots. To control the false-discovery rate across all tested ASVs, p-values were converted to FDR-values. An FDR threshold of q < 0.05 was considered statistically significant unless stated otherwise. After the differentially abundant taxa (*p* ≤ 0.05) that best explained the differences between the two participant groups were identified, a linear discriminant analysis (LDA) effect size (LEfSe) approach was applied to obtain an LDA-based effect size score^18^. The R package pheatmap was used to identify differential expression of ASVs among samples. Core microbiome detection is very important for understanding the stable, consistent elements across complex microbial assemblages. A core is often defined as the suite of members shared across microbial consortia from similar habitats and is represented by the Heatmap, ASVs with prevalence ≥ 85% (is chosen cut-off) are designated “core”. Finally, Spearman rank correlation analysis was performed to determine the bacterial associations.

## Results

### Patient demographics

A total of 35 participants were enrolled in the study (Table A6 supplementary); all of them were males aged 50–80 years, non-smokers, free of comorbidity, and weren’t on any antibiotics or corticosteroids for the past three months. The samples were assigned to two clinical states: S-COPD (*n* = 18) and AE-COPD (*n* = 17).

### Sequence data profile

**Fig. 1:**
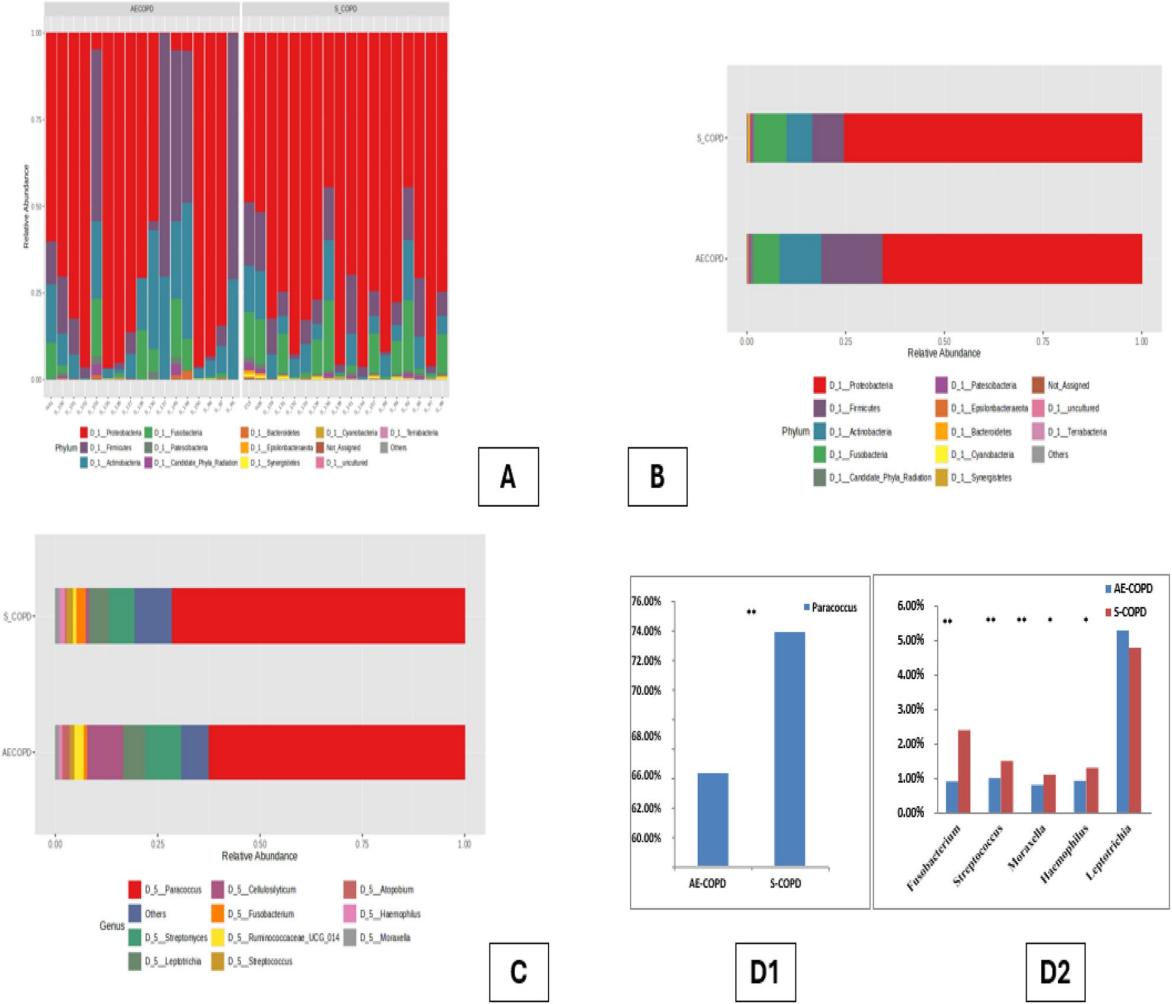
**(A**,** B)** Bar plots of the relative abundances of different phyla within the sputum microbiomes of the S-COPD and AE-COPD groups (A: in 35 samples; B: collective for the 2 groups); (**C**) Bar plots of the relative abundance of the most prevalent genera in the sputum microbiome of the S-COPD and AE-COPD groups; (**D1 and D2**) Bar plots of significant relative abundance of the most abundant genera between S-COPD and AE-COPD groups, using Chi-Square *P* ≤ 0.01**, ≥ 0.01* (**D1**: *Paracoccus* relative abundance; **D2**: relative abundance of the most abundant genera).

The sequence data for the raw data were deposited with the accession number PRJNA1021628 in the NCBI Bioproject (http://www.ncbi.nlm.nih.gov/bioproject). There were 495 Amplicon Sequence Variants (ASVs) recognized across the 35 samples for the microbiota. The rarefaction curves confirmed that the sequenced samples covered the dominant members of the bacterial communities (Fig. 1 supplementary). The demultiplexing step of the paired-end sequences resulted in a total of 9,351,510 reads, with a minimum of 127 and a maximum of 428,097.

### The sputum microbiome taxonomic profile

The entire taxonomic profile of the two studied groups included 13 phyla, 21 classes, 29 orders, 41 families, and 62 genera (Table A1 Supplementary).

#### Relative abundance of microbial communities in S-COPD and AE-COPD

The microbial composition in sputum was similar in patients in both groups, with slight differences. At the phylum level, the downstream analysis revealed that Proteobacteria was the most abundant phylum in most samples within the two groups and was significantly more prevalent in the S-COPD group (S-COPD: 75%, AE-COPD: 66%, *p* = 0.0027). Additionally, Fusobacteria was more abundant in the S-COPD group (S-COPD: 8%, AE-COPD: 6%, *p* = 0.023). On the other hand, Firmicutes and Actinobacteria were more abundant in the AE-COPD group than in the S-COPD group, where Firmicutes was significantly more abundant (S-COPD: 8%, AE-COPD: 16%, *p* = 0.029) and Actinobacteria were more prevalent but with a nonsignificant difference (S-COPD: 7%, AE-COPD: 11%). (Fig. 1A and B)

At the genus level, sixty-two genera were identified among the two groups. The identified genera sorted from higher to lower abundance, the most common genera are presented in Fig. 1 C & D. *Paracoccus*,* Moraxella*,* Fusobacterium*,* Streptococcus*, and *Haemophilus* major genera showed a significant difference between the two groups, being more prevalent in S-COPD, while *Leptotrichia* was more significantly prevalent in AE-COPD (Table A2 supplementary).

**Fig. 2:**
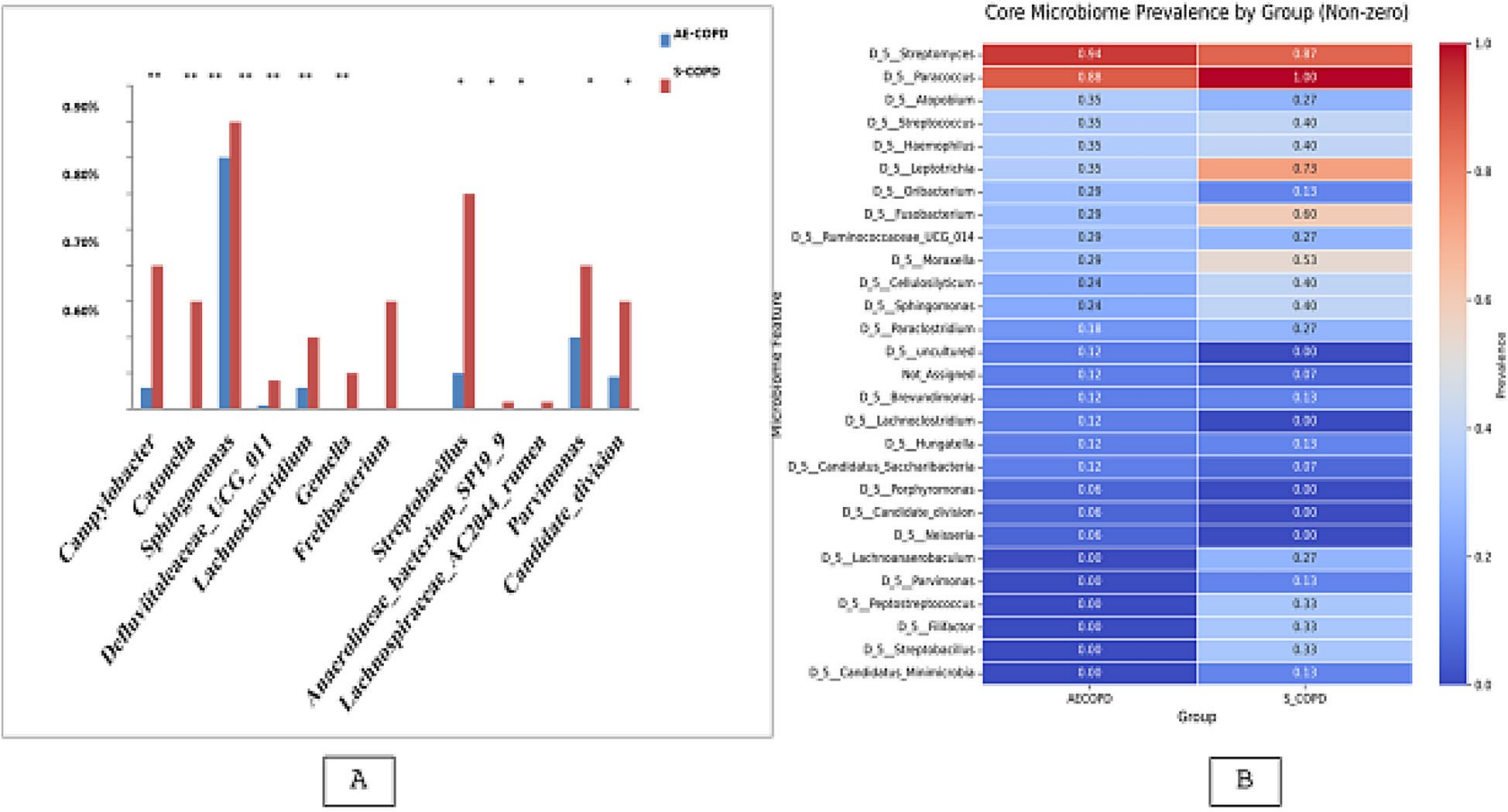
(**A**) Bar plots of significant relative abundance of minor taxa genera between S-COPD and AE-COPD groups, using Chi-Square *P* ≤ 0.01**, ≥ 0.01*; (**B**) Heat map of the core microbiome at the genus level of the sputum microbiome of the two diagnostic groups The vertical bar with values is the relative abundance key. Units = fraction of total reads (i.e. 0.94 = 94%). Genera are on left side.

 According to minor taxa (< 0.5), the relative abundance of the following genera were very significantly higher in S-COPD compared to AE-COPD like *Campylobacter* (*p* = 0.0009), *Catonella* (*p* = 0.001), *Sphingomonas* (*p* = 0.0018), *Defluviitaleaceae_UCG_011*(*p* = 0.0032), *Lachnoclostridium* (*p* = 0.0051), *Gemella* (*p* = 0.0052) and *Fretibacterium*(*p* = 0.0053) while *Streptobacillus* (*p* = 0.02), *Anaerolineae* (*p* = 0.022), *Lachnospiraceae_AC2044* (*p* = 0.022), *Lysinibacillus* (*p* = 0.022), *Candidate_Absconditabacteria* (*p* = 0.023*)*,* Erythrobacter* (*p* = 0.023), *Parvimonas* (*p* = 0.025) and *Candidatus_division* (*p* = 0.027) showed low significant difference (Fig. 2 A).

#### Core microbiome

The core microbiome analysis was conducted to identify the stable and consistently present taxa within diverse microbial communities. Our data revealed that we had 62 genera. The genus considered unique represented the minority of the founded genera, which were 6 genera (9.5%) detected in only one sample and 5(8%) genera among the AE-COPD group, while 9 genera were detected in only one sample (14.5%) and 17 (27.4%) genera among the S-COPD group (Table A3 supplementary).

The core genera were 2 genera (*Streptomyces* and *Paracoccus*, 3.2%) common in both groups while there were 6 other genera present in all samples in the S-COPD group (S*treptococcus*,* Fusobacterium*,* Leptotrichia*,* Moraxella*,* Oribacterium* and *Shingomonas*) as shown in Fig. 2B. Most of the detected genera were distributed genera that may be present in some but not all the samples.

#### Bacterial diversity analysis

##### Alpha diversity

**Fig. 3:**
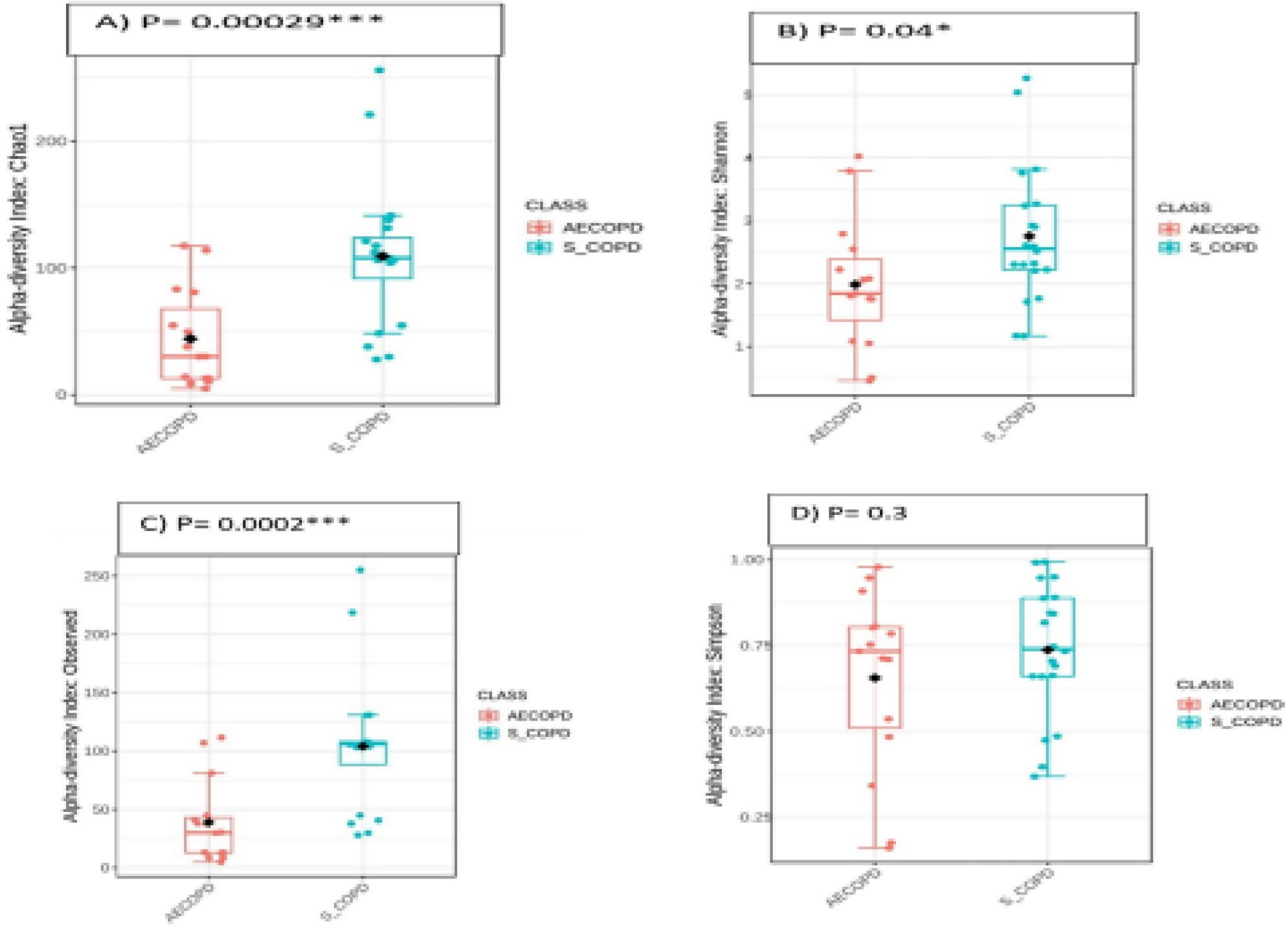
Boxplot presenting the alpha diversity in terms of (**A**) Chao1, (**B**) Simpson’s, (**C**) observed and (**D**) Shannon’s diversity indices. Differences between S-COPD and AE-COPD were evaluated via the Mann‒Whitney samples t test at the 0.05 level.

Different alpha diversity indices were calculated and revealed greater diversity for stable patients than for exacerbated patients in terms of the Chao1, Simpson, observed, and Shannon diversity indices (Fig.  3A, B, C, &D). The results revealed a significant difference between the S-COPD group and the AE-COPD group according to the Chao, Shannon’s, and observed indices (P values equal to 0.00029, 0.04, and 0.0002, respectively), whereas Simpson’s indices were not significantly different (*, *p* = 0.03*), as revealed by the ANOVA samples test and Mann‒Whitney test, respectively.

### Beta diversity

When comparing the respiratory microbiome of S-COPD patients against AE-COPD, compositional variations were significantly recorded in the overall microbial structure (Beta diversity). Three diversity metrics were calculated: Bray-Curtis, weighted UniFrac distance, and unweighted UniFrac distance.

**Fig. 4:**
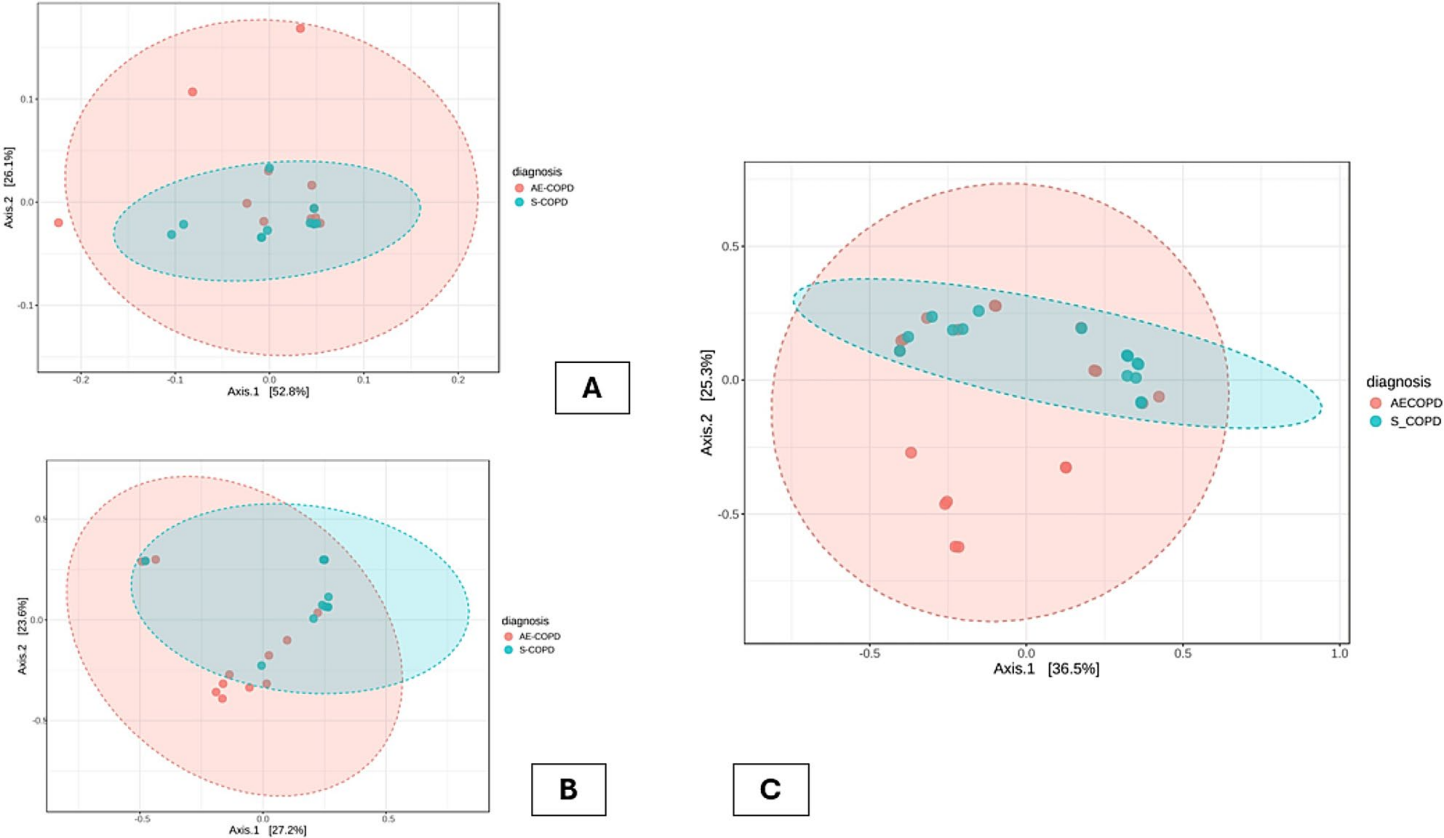
(**A**) PCoA 2D, beta diversity between AE-COPD and S-COPD based on weighted UniFrac with PREMANOVA as the statistical method, p value = 0.354; (**B**) PCoA 2D, beta diversity between AE-COPD and S-COPD based on unweighted UniFrac with PREMANOVA as the statistical method, p value = 0.009; (**C**) PCoA 2D, beta diversity between AE-COPD and S-COPD based on Bray‒Curtis distance with PREMANOVA as the statistical method, p value = 0.02.

Beta diversity significant measures were performed using the test PERMANOVA to compare the bacterial composition among the sample microbiota from S-COPD and AE-COPD individuals. Clustering analysis of the data using PCoA was performed on the distance metrics of the biome tables. PCoA visualization plot of weighted UniFrac distance showed high clustering according to the S-COPD of the subject, whereas AE-COPD appeared more scattered (where PC1 variability = 52.8%, PC2 = 26.1% and PC3 = 8. %) Fig. 4A . While the PCoA visualization plot of unweighted UniFrac distance showed high clustering for both the Stable state and exacerbated (where PC1 variability = 27.7%, PC2 = 23.6% and PC3 = 15%), Fig. 4B. Moreover, the PCoA plot of Bray-Curtis showed that S-COPD were highly clustered, while the AE-COPD were more disseminated (PC1 = 36.5%, PC2 = 25.3% and PC3 = 15.1%), Fig. 4C. Statistical analysis was carried out using PREMANOVA, and the p-values were as follows: S-COPD vs. AE-COPD *p* = 0.354, 0.009, 0.02 for weighted UniFrac, unweighted UniFrac, and Bray-Curtis, respectively. As a result, S-COPD and AE-COPD were significantly distinguished by Bray-Curtis and unweighted UniFrac distances (PERMANOVA *p* = 0.020 and 0.009, respectively), but not by weighted UniFrac (*p* = 0.354).

### LEfSe analysis

**Fig. 5:**
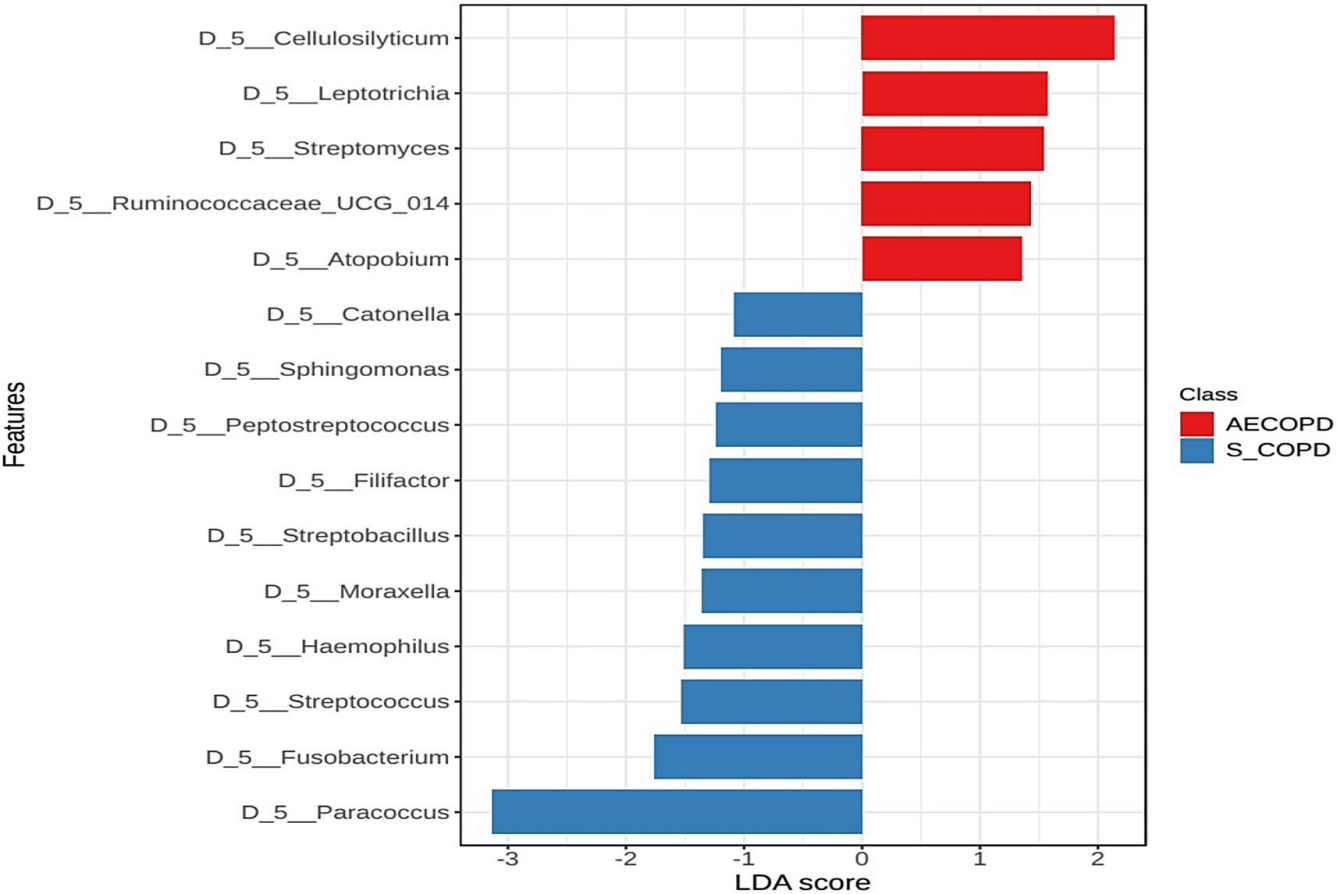
Histogram of linear discriminant analysis (LDA) effect size (LEfSe) at the genus level in sputum samples from the S-COPD group and the AE-COPD group, with a p-value cutoff of 0.05. Negative (blue bars) LDA scores represent bacterial groups overrepresented in S-COPD patients, whereas positive (red bars) scores represent bacterial groups overrepresented in AE-COPD patients.

LEfSe on an LDA score of ± 3 at a p value cutoff of 0.05 and false discovery rate (FDR) adjustment revealed significant differences between the microbial populations of S-COPD and AE-COPD patients in terms of the abundance of *Paracoccus* (LDA score = 3.14), *Fusobacterium* (LDA score = 1.81), *Streptococcus* (LDA score = 1.53), *Haemophilus* (LDA score = 1.51), *Moraxella* (LDA score = 1.36) and *Streptococcus* (LDA score = 1.35) genera in the S-COPD group, which were greater than those in the AE-COPD group (Fig. 5).

### Clustering and correlation analysis

#### Clustering

**Fig. 6:**
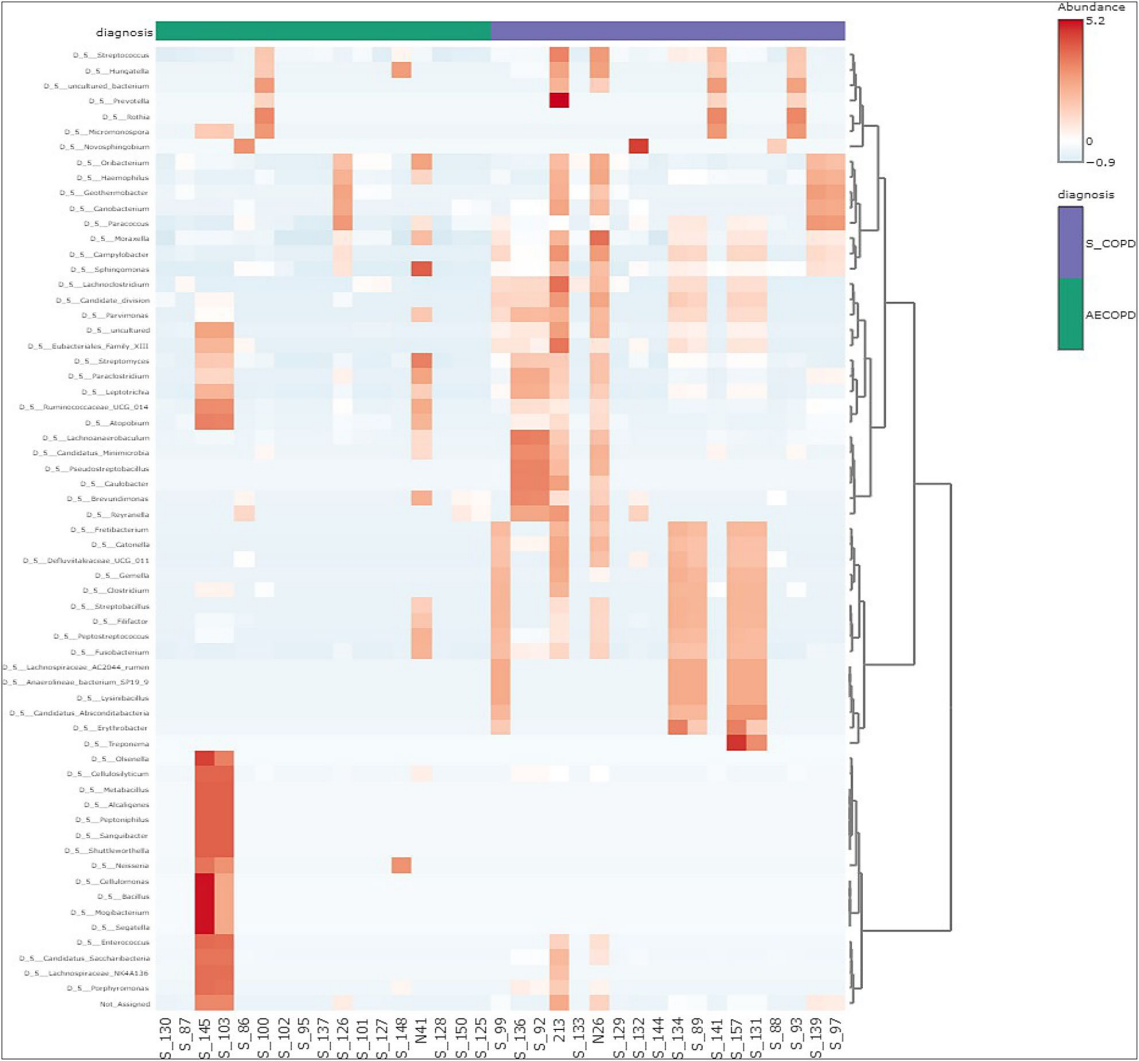
Heat map with dendrogram at the genus level using a gradient heat map. 62 genera were used in hierarchical clustering between S-COPD and AE-COPD. The darker the red color, the more predominant genus FDR of q- value ≤ 0.05 is considered significant.

The 62 genera were used in hierarchical clustering to evaluate the relationships between two groups (S-COPD, AE-COPD) using weighted pair clustering based on Bray-Curtis measurements. Each individual carried a relatively specific bacterial community as represented in Fig. 6.

#### A correlation between S-COPD and AE-COPD community members

**Fig. 7:**
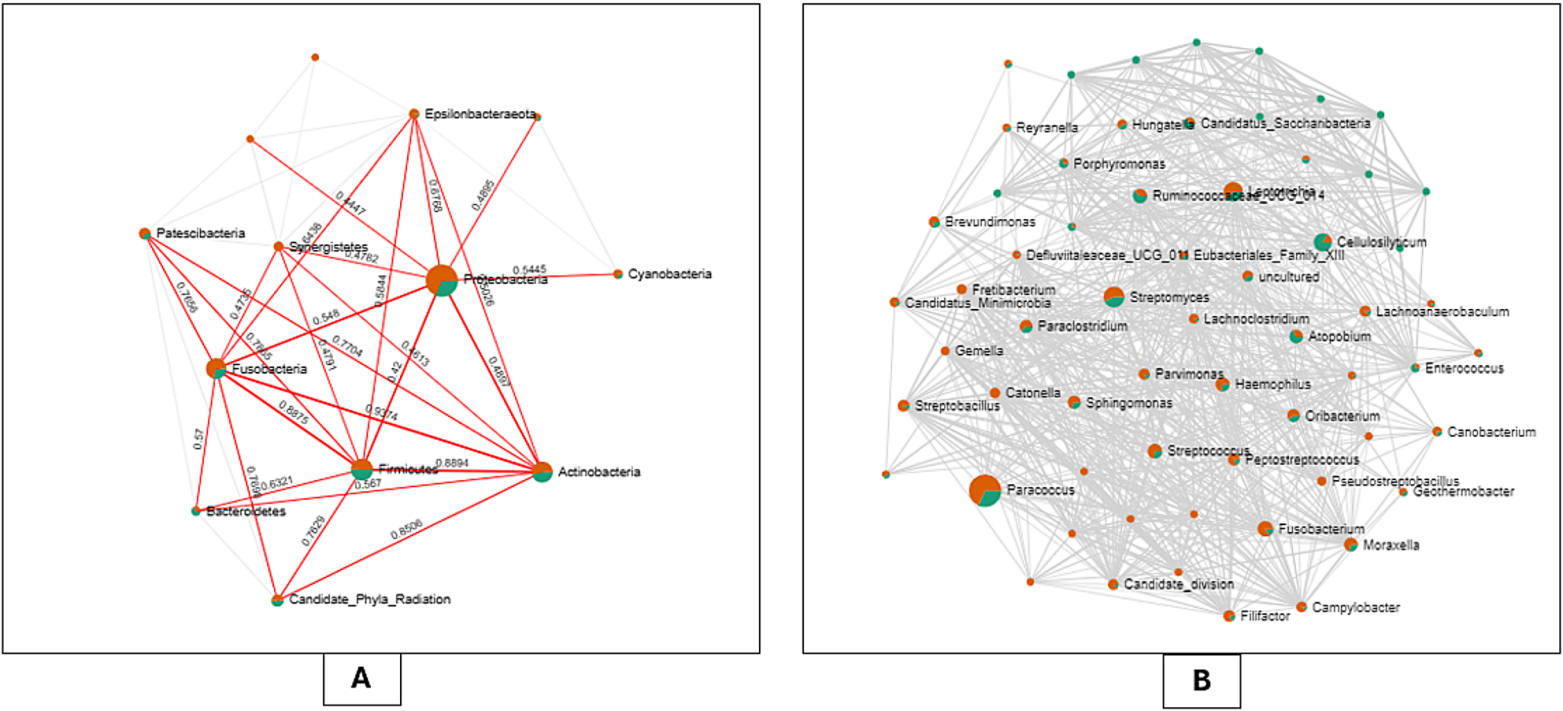
(**A**) Bacterial network analysis based on Spearman rank correlation at the phylum level according to diagnostic stage. The average abundance of a genus is represented by the size of the circle, the correlation between two species is represented by a line, the strength of the connection is represented by the line’s thickness, and a positive correlation is represented by the line’s orange color, Green nodes = taxa whose relative abundance is positively correlated and Orange nodes = taxa whose relative abundance is negatively correlated; (**B**) Bacterial network analysis based on Spearman rank correlation at the genus level according to diagnostic stage. (correlation lines that don’t appear colored is a non-significant correlation).

For understanding the interactions that take place across different bacterial populations, correlation analysis was performed. The Spearman correlation coefficient was used to determine the correlation between different bacterial ASVs at different taxonomic levels. Among the highest abundance phyla, there was a significant correlation found between ASVs within phylum Bacteroidetes versus Actinobacteria and Fusobacteria phylum (*r* = 0.7). There was a significant positive correlation between members of the Firmicutes phylum vs. Proteobacteria and Synergistetes (*r* = 0.5). Also, there was a significant positive correlation between members of phylum Fusobacteria vs. Proteobacteria and Synergistetes (*r* = 0.6, 0.5, respectively), (Fig. 7A &Table A4 supplementary).

At the genus level, significant positive correlations were detected between ASVs assigned to the genera *Atopobium*,* Haemophilus*,* Paracoccus* and *Streptococcus* and *Streptomyces* (*r* = 0.45, 0.33, 0.52 and 0.4, respectively), and significant positive correlations were detected between ASVs belonging to the genera *Fusobacteria*,* Haemophilus*,* Leptotrichia*,* Moraxella* and *Ruminococcaceae_UCG_014* and the genus *Streptococcus* (*r* = 0.36, 0.42, 0.41, 0.35 and 0.34, respectively). A significant positive correlation was detected between ASVs belonging to the genera *Leptotrichia* and *Paracoccus* (*r* = 0.5), *Haemophilus* and *Leptotrichia* (*r* = 0.38) and *Fusobacteria* and *Haemophilus* (*r* = 0.57), and a negative correlation was detected between *Neisseria* and *Paracoccus* (*r*= -0.30) (Table A5 & Fig. 7B).

###  Discussion

 Research on the COPD microbiome has revealed its role in both disease development and exacerbation, and the disordered microbiome is believed to be involved in the pathogenesis of COPD by modulating inflammatory and immune responses^19^. In our study, regarding the overall phyla composition, the most prevalent phyla in the two studied groups were Proteobacteria, Fusobacteria, Firmicutes, and Actinobacteria. Proteobacteria was the most abundant phylum in most samples within the two groups and was significantly more prevalent in the S-COPD group (*p* = 0.0027). Generally, different COPD microbiome studies have identified Proteobacteria, Firmicutes, Bacteroides and Actinobacteria as the most abundant respiratory microbiome phyla; however, one of them is the most dominant phylum. In accordance with our results, *Garcia-Nunez* et al. revealed the predominance of the Proteobacteria, Firmicutes, and Actinobacteria phyla^20^. Another study reported that the Proteobacteria phylum constituted half of the microbiome of COPD subjects, where Firmicutes were dominant in the healthy microbiome^21^. Many previous studies were in line with our findings about the predominance of the Proteobacteria phylum (22.23.24). Yan et al.^25^ performed metabolomic and transcriptomic profiling of sputum samples comparing patients with COPD and healthy control subjects. They suggested that the COPD microbiome fosters amino acid anabolism relative to the healthy respiratory microbiome. They also found that altered tryptophan metabolism and depletion of indole-3-acetic acid may promote airway neutrophilia and increase epithelial cell apoptosis. This was in accordance to the study done by Dicker et al.^26^ who related the predominance of bacterial members from the Proteobacteria phylum in COPD respiratory microbiome to neutrophil activation in the lower respiratory tract and impaired lung function.

In contrast to our observations, other studies have reported a greater abundance of Firmicutes in COPD patients (27.28); for example, a study performed by Goolman et al. revealed the dominance of four phyla, Firmicutes, Proteobacteria, Bacteroides and Actinobacteria, with an increased relative abundance of Firmicutes. Wang et al. revealed that Firmicutes was the most dominant phylum in the sputum microbiome of COPD patients, followed by Actinobacteria and Proteobacteria.

The dissimilarity in the predominant phyla between different studies could be attributed to many factors, including different sample sizes; different patient sample groups (e.g., smokers, non-smokers, and types of treatment); different sample types (e.g., sputum, bronchoalveolar lavage or tracheal aspirate); different techniques; and other general factors, such as geographical location, weather, air pollution, and other environmental conditions.

At the genus level, an increase in the relative abundance of a certain genus may be conceived as a cause of COPD exacerbation, but it may differ from the culture results, as colonizing microorganisms may be cultivated easily from bronchial samples, as reported for patients with exacerbation showing chronic *Pseudomonas aeruginosa* colonization (29. 30). The genera most frequently reported to be predominant in different previous COPD studies were *Haemophilus*,* Veillonella*,* Prevotella*,* Rothia*,* Lactobacillus*,* Granulicatella Staphylococcus* and *Streptococcus* (21.27,28). Notably, our study revealed the predominance of some of these genera, although the abundance of *Paracoccus*, which represented the most abundant genus in our study with increased relative abundance stable states, was very unique. The genus *Paracoccus* is environmental pathogen found in in soil and brines. *Paracoccus yeei* has been identified as a human pathogen as it possesses specific virulent genes. Infections with this opportunistic bacterium are usually reported in immunocompromised patients^31^. Identification of *Paracoccus* in these samples may reflect upper-airway contamination, geographic factors, or sampling bias rather than true lower-airway colonization. This finding needs further study to validate whether *Paracoccus*, may have a role in pathogenicity or was it a contaminant from the environment.

We estimated a low degree of similarity between the core microbiome for each of the two stages of disease (stable and exacerbation). Two ASVs were present in all the subjects; these belong to the genera *Streptococcus*, and *Streptomyces*. Gupta et al. reported that ten ASVs were shared among two stages of disease (stable and exacerbation), belonging to the genera *Oribactrum*, *Streptococcus*, and *Sphingomonas*^29^. Einarsson et al. estimated that the co-occurrence of bacterial taxa and the observation of a putative ‘core’ community within the lower airways, including *Prevotella* spp., *Veillonella* spp. and *Actinomyces* spp., were also apparent in their study^30^.

Our results revealed that microbiome diversity was lower in AE-COPD patients than in S-COPD patients. This aspect is distinct among different studies, and some studies have shown an overall reduction in microbial diversity during COPD exacerbations compared with stable patients^6,32 and 33^. These results are consistent with those of the present study, thus suggesting that microbial diversity may be a biological indicator of AE-COPD. Garcia-Nuñez et al. reported decreased alpha diversity in advanced COPD patients compared with moderate-to-severe disease patients^20^. These findings support the occurrence of severity-related changes in the bronchial microbiome in COPD patients. Sze et al. found that α-diversity, especially richness, declines in patients with increased airflow limitation and emphysema^34^.

Our results do not correlate with previous data from Pragman and colleagues, who did not find severity-related differences in microbial diversity in BALF samples from COPD patients^22^. Hazra et al. reported that the alpha diversity of moderate COPD patients was lower than that of patients with severe COPD^35^. However, Goolam et al. did not observe a significant difference in microbial diversity between stable COPD and exacerbated COPD groups^27^. Jubinville et al. reported a difference in alpha diversity when paired samples were compared, i.e., the diversity in the paired samples differed across the disease state, with most exacerbated samples showing greater diversity^36^.

This inconsistency among studies could be due to distinct sampling methods, COPD states and exacerbations, different geographic regions and diversity measures used, whereas Jubinville et al. used the Simpson index. Unlike the Shannon index, the Simpson index is more affected by the relative abundances (i.e., evenness) of the species in a sample; this suggests that during the exacerbated state of disease, the abundances of species/ASVs change but not the number of species/ASVs (richness)^37^. Additionally, the type of treatment used in COPD patients is another factor affecting microbiome composition. Data show that the use of antibiotics and corticosteroids in exacerbated COPD patients influences the respiratory microbiome^38^. Inhaled corticosteroids, the main treatment for COPD, significantly increase bacterial burden and diversity, with increased pathogenic strains^39,40,41^. Antibiotics are usually used in COPD patients with frequent exacerbations to decrease the recurrence of these episodes. This type of therapy was found to decrease microbial diversity, which lasts for months and persists during stable periods between episodes^42,43^.

Principal coordinate analysis (PCoA) revealed significant differences in the microbial community structure between the AE-COPD and S-COPD patients (*p* = 0.02). This pattern suggests that rather than a quantifiable rearrangement of the abundant taxa that dominate the weighted metric, the problem is linked to changes in community composition (presence/absence of low-abundance ASVs).This finding was in accordance with the findings of Su et al., who reported that principal coordinate analysis (PCoA) revealed significant differences in microbial community structure between the AECOPD group and stable group (*p* = 0.02) and between the AECOPD group and healthy control group (*p* = 0.035)^33^. Goolam et al. and colleagues reported that beta diversity measures showed no clustering for any of the variables via PCoA or weighted UniFrac (for microbiome) measures^27^.

In contrast to our results, Gupta et al., PCoA revealed extensive overlap in membership between the bacterial communities of the ECOPD and stable COPD disease groups^29^. This inconsistency among studies could be due to the use of different distance methods.

In addition, the significant effect found by Bray-Curtis and unweighted UniFrac but not by weighted UniFrac suggests that while the quantitative structure of the dominant community stays constant, COPD exacerbation is followed by a turnover in the rare biosphere, i.e., gain or loss of minor ASVs. Biologically, this suggests that the airway ecosystem during acute exacerbation is more sensitive to invasion or extinction of rare populations than to wholesale restructuring of dominant taxa. On the other hand, the lack of a weighted-UniFrac signature supports a concept in which community-wide dysbiosis distinguishes AE-COPD from the stable state, arguing against a single or a few abundant taxa drastically increasing during exacerbation.

 LEfSe analysis at the genus level revealed that the sputum microbiome of the AE-COPD group was characterized by a dominance of *Cellulosilyticun*,* Liptotrichia* and *Streptomyces*, whereas the microbiome in the stable COPD group was dominated by the genera *Paracoccus*, *Fusobacterium*, *Streptococcus Haemophilus* and *Moraxella (p* < 0.05).

 Hazra et al. reported that six marker genera, *Streptococcus* and *Rothia*, were highly abundant in moderate COPD patients, whereas *Leptotrichia* and *Pseudomonas* were highly prevalent in patients with severe COPD^35^. Haldar et al. reported that linear discriminant effect size (LEfSe) analysis revealed that a greater abundance of Proteobacteria and lower proportions of Firmicutes, Bacteroidetes and Actinobacteria were the major contributors to the differentiation of COPD patients from healthy controls^21^. Chang et al. reported that LEfSe analysis revealed that, during the AECOPD period, *Pseudomonas* was highly abundant. In stable COPD, *Haemophilus influenzae* and *Pasteurellales* are more abundant^44^.

 The Spearman correlation coefficient was used to determine the positive correlation between different bacterial ASVs at different phylum and genus levels. Most of the genera in stable COPD and AECOPD patients were more positively correlated. We also noted that many potentially clinically relevant taxa, such as *Streptococcus* and *Haemophilus*, were correlated with other taxa. This finding was consistent with those of other studies^23,29^. In contrast, Yang et al. reported a negative correlation between ASVs assigned to the genus *Haemophilus* and *Streptococcus* in non-frequent exacerbations, and Li et al. reported a negative correlation between ASVs assigned to the genus *Fusobacteria* and *Streptococcus* and *Fusobacteria* and *Streptococcus* in stable COPD patients^45,46^. However, correlations between taxa are not proof of functional relationships between members of the community. Therefore, further studies are needed to focus on the functional role of such taxa found within these communities.

###  Study limitations

The noteworthy limitations of our study are as follows: the sample size was small, and the samples were cross-sectional rather than longitudinal; thus, the interference of coexisting factors was unavoidable, and there was a lack of healthy control subjects. Sputum samples were used in this study; although they are the most commonly used samples in airway microbiome studies (noninvasive methods), they have the drawbacks of being a mixture of upper and lower respiratory tract microbiomes. In addition, “unique” ASVs may be depth artefacts as these samples had reached a plateau on the rarefaction curve (Figure S1, supplementary). Regarding patients’ demographic data, we acknowledge that female participants were excluded from this study, which is contrary to established guidelines such as the SAGER guidelines that advocate for the inclusion of all sexes and genders to ensure comprehensive scientific representation. Our decision to include only male patients was primarily driven by the fact that the majority of COPD cases during the study period were male, reflecting a local epidemiological pattern. Consequently, the number of female patients was too limited to permit meaningful statistical analysis or subgroup comparisons. Furthermore, comprehensive clinical parameters, such as complete blood count and pro-inflammatory cytokine profiles, were not included in the demographic data. This is because the study population consisted of outpatients who did not routinely undergo blood investigations unless clinically indicated by the treating physician.

##  Conclusion

 In conclusion, we found that dysbiosis in the lung microbiome among different COPD states is characterized by a high abundance of Proteobacteria, especially those associated with the *Paracoccus* microbiome. However, the precise role of this dysbiosis in the pathogenesis and severity of COPD is still unclear. More evidence is needed to suggest that this taxon may be more likely to be found in the lungs of Egyptian COPD patients due to geographic differences. More studies to validate this finding, including larger, geographically diverse cohorts, longitudinal analyses, and the integration of metagenomic and culture-based approaches. In addition, applying new metagenomics and bioinformatics analyses and metabolomics are necessary to determine the role of the respiratory microbiome and its metabolic profile in COPD progression and open future avenues to apply these findings in clinical practice to introduce new therapeutic approaches and improve clinical outcomes.

## Electronic Supplementary Material

Below is the link to the electronic supplementary material.

Supplementary Material 1

## Data Availability

sequence data that support the findings of this study have been deposited in the SRA with the primary accession code PRJNA 1021628 in the NCBI Bioproject ( http:/www.ncbi.nlm.nih.gov/bioproject ).
