# Supplementary material for: Comparative analysis of the sputum microbiota in different COPD clinical states
Source: Sci Rep. 2026 Jun 8;16:17694. doi: 10.1038/s41598-026-53780-1 (PMC13246853; doi:10.1038/s41598-026-53780-1)
Supplement: Supplementary file 1 — Supplementary Material 1 [file 41598_2026_53780_MOESM1_ESM.docx]

**Supplementary** **methods and statistics:**

A negative control was applied from the starting of DNA extraction till the end of the sequencing process. In addition, all samples and negative control were extracted for DNA and PCR at our lab at the same time, then the DNA samples were shipped to Italy under stated conditions by the IGA Technology Services Company (Udine, Italy). The original no. of shipped samples was 47 and after QC, we completed our analysis on 35 and excluded bad samples.

Sequences that were of poor quality were trimmed using the parameter of the QIIME script split_libraries.py (minimum average quality score equals 25, minimum/maximum sequence 57 length equals 200/1000 base pairs, zero ambiguous base calls, and zero mismatches in the primer sequence) Quality filtering and removal of reads 220 < and > 299.

Quality control checks (Fast QC) were performed before analyzing these sequences to draw biological conclusions, to ensure that the raw data looked good and that there were no problems or biases in the data.

The average quality score of full-length forward reads was 38 at the 50 percentile, which was higher than the reverse reads' 34 at the 50 percentile. The average length of approximately 460 bases was obtained to determine the approximate phylogenetic position of sequences.

A final data set of 113,990 out of 966,140 sequence reads was obtained after excluding low-quality sequence reads, denoising, and chimeras removal**.** The truncation length parameter of DADA2 was ptrunc-len-f 280 for forward reads and p-trunc- len-r 240 for reverse reads, with an average quality score of 21. We added table A7 in the supplementary for QC of samples.

**Table A1: Relative abundance of different classes within respiratory microbiome between S-COPD and AE-COPD**

| **Name** | | **AE-COPD** | | **S-COPD** | | **P-value** | | **FDR** | |
| --- | --- | --- | --- | --- | --- | --- | --- | --- | --- |
| Campylobacteria | | 0.06% | | 0.4% | | 0.0003*** | | 0.01** | |
| Alphaproteobacteria | | 63.7% | | 72.9% | | 0.003** | | 0.02* | |
| Gammaproteobacteria | | 1.7% | | 2.3% | | 0.004** | | 0.02* | |
| WWE3 | | 0% | | 0.07% | | 0.0056 | | 0.02* | |
| Synergistia | | 0% | | 0.3% | | 0.005** | | 0.02* | |
| Saccharimonadia | | 0.1% | | 0.4% | | 0.007** | | 0.02* | |
| Chloroflexota | | 0% | | 0.02% | | 0.02* | | 0.04* | |
| Fusobacteriia | | 6.3% | | 8% | | 0.02* | | 0.04* | |
| Bacilli | | 1.2% | | 1.6% | | 0.02* | | 0.04* | |
| Clostridia | | 14.3% | | 6.4% | | 0.03* | | 0.04* | |
| Actinobacteria | | 8.9% | | 6.2% | | 0.05 | | 0.07 | |
| Betaproteobacteria | | 0.05% | | 0% | | 0.07 | | 0.1 | |
| Candidatus | | 0.8% | | 0.4% | | 0.09 | | 0.1 | |
| Spirochaetia | | 0% | | 0.02% | | 0.2 | | 0.2 | |
| Bacterium | | 0.02% | | 0.03% | | 0.2 | | 0.2 | |
| Cyanophyceae | | 0.1% | | 0.2% | | 0.2 | | 0.3 | |
| Deltaproteobacteria | | 0.042% | | 0.062% | | 0.4 | | 0.5 | |
| Actinomycetes | | 0.052% | | 0.02% | | 0.5 | | 0.7 | |
| Bacteroidia | | 0.2% | | 0.07% | | 0.6 | | 0.7 | |
| Coriobacteriia | | 1.9% | | 0.5% | | 0.7 | | 0.8 | |
| Gracilibacteria | | 0.4% | | 0.06% | | 0.8 | | 0.9 | |
| Not_Assigned | | 0.09% | | 0.07% | | 0.9 | | 0.9 | |

AE- COPD: exacerbated COPD; S- COPD: Stable COPD; FDR: False Discovery Rate; *significant

**Table A2: relative abundance of different genera within the respiratory microbiome between the S-COPD and AE-COPD groups**

| **Name** | **AE-COPD** | **S-COPD** | **P-value** | **FDR** | | |
| --- | --- | --- | --- | --- | --- | --- |
| *Campylobacter* | 0.06% | 0.4% | 0.0009*** |  | 0.03* |  |
| *Catonella* | 0% | 0.3% | 0.001** |  | 0.03* |  |
| *Sphingomonas* | 0.7% | 0.8% | 0.001** |  | 0.03* |  |
| *Defluviitaleaceae_UCG_*  *011* | 0.01% | 0.08% | 0.003** | 0.03* | | |
| *Paracoccus* | 62.6% | 71.6% | 0.003** |  | 0.03* |  |
| *Moraxella* | 0.8% | 1.1% | 0.003** |  | 0.03* |  |
| *Fusobacterium* | 0.9% | 2.4% | 0.004** |  | 0.03* |  |
| *Lachnoclostridium* | 0.06% | 0.2% | 0.005** |  | 0.03* |  |
| *Gemella* | 0% | 0.1% | 0.005** |  | 0.03* |  |
| *Fretibacterium* | 0% | 0.3% | 0.005** |  | 0.03* |  |
| *Streptococcus* | 1.1% | 1.5% | 0.007** |  | 0.03* |  |
| *Haemophilus* | 0.92% | 1.3% | 0.01* |  | 0.04* |  |
| *Streptobacillus* | 0.1% | 0.6% | 0.02* |  | 0.04* |  |
| *Anaerolineae_bacterium_*  *SP19_9* | 0% | 0.02% | 0.02* | 0.04* | | |
| *Lachnospiraceae_AC204*  *4_rumen* | 0% | 0.02% | 0.02* | 0.04* | | |
| *Lysinibacillus* | 0% | 0.01% | 0.02* | 0.04* | | |

| *Candidatus_Absconditab acteria* | 0% | 0.01% | 0.02* | 0.04* |
| --- | --- | --- | --- | --- |
| *Erythrobacter* | 0% | 0.001% | 0.02* | 0.04* |
| *Parvimonas* | 0.2% | 0.4% | 0.02* | 0.04* |
| *Candidate_division* | 0.09% | 0.3% | 0.02* | 0.04* |
| *Leptotrichia* | 5.3% | 4.8% | 0.03* | 0.04* |
| *Eubacteriales_Family_XI II* | 0.06% | 0.08% | 0.04* | 0.08 |
| *Caulobacter* | 0% | 0.05% | 0.04* | 0.1 |
| *Pseudostreptobacillus* | 0% | 0.2% | 0.04* | 0.1 |
| *Peptostreptococcus* | 0.3% | 0.6% | 0.04* | 0.1 |
| *Uncultured* | 0.3% | 0.3% | 0.05 | 0.1 |
| *Streptomyces* | 8.8% | 6.2% | 0.05 | 0.1 |
| *Clostridium* | 0.01% | 0.03% | 0.06 | 0.1 |
| *Neisseria* | 0.05% | 0% | 0.07 | 0.1 |
| *Paraclostridium* | 0.8% | 0.7% | 0.08 | 0.1 |
| *Filifactor* | 0.2% | 0.6% | 0.09 | 0.1 |
| *Lachnoanaerobaculum* | 0.2% | 0.5% | 0.1 | 0.2 |
| *Candidatus_Minimicrobi a* | 0.07% | 0.2% | 0.1 | 0.2 |
| *Ruminococcaceae_UCG_ 014* | 2.2% | 0.7% | 0.1 | 0.2 |
| *Not_Assigned* | 0.5% | 0.2% | 0.1 | 0.2 |
| *Alcaligenes* | 0.02% | 0% | 0.1 | 0.2 |
| *Metabacillus* | 0% | 0.001% | 0.1 | 0.2 |
| *Peptoniphilus* | 0% | 0.001% | 0.1 | 0.2 |
| *Sanguibacter* | 0.03% | 0% | 0.1 | 0.2 |
| *Shuttleworthella* | 0.03% | 0% | 0.1 | 0.2 |
| *Bacillus* | 0.001% | 0% | 0.1 | 0.2 |
| *Cellulomonas* | 0% | 0.001% | 0.1 | 0.2 |
| *Mogibacterium* | 0% | 0.001% | 0.1 | 0.2 |
| *Olsenella* | 0% | 0.001% | 0.1 | 0.2 |
| *Segatella* | 0% | 0.001% | 0.1 | 0.2 |
| *Treponema* | 0% | 0.001% | 0.1 | 0.2 |
| *Hungatella* | 0.2% | 0.2% | 0.1 | 0.2 |
| *Oribacterium* | 0.8% | 0.6% | 0.1 | 0.2 |
| *Canobacterium* | 0.1% | 0.2% | 0.2 | 0.2 |
| *uncultured_bacterium* | 0.03% | 0.06% | 0.2 | 0.2 |
| *Candidatus_Saccharibact eria* | 0.7% | 0.2% | 0.2 | 0.3 |
| *Reyranella* | 0.03% | 0.07% | 0.3 | 0.3 |
| *Prevotella* | 0.01% | 0.01% | 0.3 | 0.3 |
| *Geothermobacter* | 0.04% | 0.06% | 0.3 | 0.4 |
| *Brevundimonas* | 0.3% | 0.4% | 0.4 | 0.4 |
| *Lachnospiraceae_NK4A1 36* | 0.08% | 0.01% | 0.4 | 0.5 |
| *Cellulosilyticum* | 9% | 1% | 0.6 | 0.6 |
| *Rothia* | 0.02% | 0.02% | 0.6 | 0.6 |
| *Novosphingobium* | 0.01% | 0.01% | 0.6 | 0.6 |
| *Micromonospora* | 0.03% | 0.01% | 0.6 | 0.7 |
| *Atopobium* | 1.9% | 0.5% | 0.7 | 0.7 |
| *Porphyromonas* | 0.2% | 0.05% | 0.8 | 0.8 |
| *Enterococcus* | 0.2% | 0.03% | 0.9 | 0.9 |

AE- COPD: exacerbated COPD; S- COPD: Stable COPD; FDR: False Discovery Rate; *significant

**Table A3: Bacterial genera that are unique in two groups**

| **Unique genera of S- COPD** | *Anaerolineae_bacteriu m_SP19_9 Candidatus_Abscondita bacteria*  *Catonella Caulobacter Fretibacterium Gemella*  *Lachnospiraceae_AC20 44_rumen Lysinibacillus Pseudostreptobacillus Erythrobacter Metabacillus Peptoniphilus Cellulmonas Mogibacterium Olsenella*  *Segatella*  *Treponema* |
| --- | --- |
| **Unique genera among S-COPD samples** | *Lachnospiraceae_NK 4A136*  *Erythrobacter Metabacillus Peptoniphilus Cellulmonas Mogibacterium Olsenella Segatella Treponema* |
| **Unique genera of AE-COPD** | *Alcaligenes Neisseria Sanguibacter Shuttleworthella Bacillus* |
| **Unique genera among AE- COPD samples** | *Defluviitaleaceae_ UCG*  *Novosphingobium Prevotella*  *Rothia Streptobacillus Bacillus* |

**Table A4: Correlation table between phyla in the sputum microbiome**

| **Taxon1** | **Taxon2** | **Correlation** | **P-value** |
| --- | --- | --- | --- |
| Actinobacteria | Bacteroidetes | 0.7* | 0.0001*** |
| Actinobacteria | Epsilonbacteraeota | 0.5 | 0.002 |
| Actinobacteria | Proteobacteria | 0.5 | 0.002 |
| Actinobacteria | Synergistetes | 0.5 | 0.005 |
| Bacteroidetes | Fusobacteria | 0.7** | 0.0001*** |
| Bacteroidetes | Patescibacteria | 0.7** | 0.0001*** |
| Cyanobacteria | Epsilonbacteraeota | 0.4 | 0.02 |
| Cyanobacteria | Proteobacteria | 0.5 | 0.0001*** |
| Epsilonbacteraeota | Firmicutes | 0.6* | 0.0001*** |
| Epsilonbacteraeota | Patescibacteria | 0.7* | 0.0001*** |
| Epsilonbacteraeota | Spirochaetetes | 0.3 | 0.03 |
| Epsilonbacteraeota | Terrabacteria | 0.6* | 0.0001 |
| Firmicutes | Proteobacteria | 0.5 | 0.01 |
| Firmicutes | Synergistetes | 0.5 | 0.003 |
| Fusobacteria | Proteobacteria | 0.6* | 0.0001 |
| Fusobacteria | Synergistetes | 0.5 | 0.004 |
| Patescibacteria | Terrabacteria | 0.5 | 0.003 |
| Proteobacteria | Synergistetes | 0.5 | 0.003 |
| Proteobacteria | Terrabacteria | 0.4 | 0.007 |
| Spirochaetetes | Synergistetes | 0.5 | 0.001 |
| Spirochaetetes | Terrabacteria | 0.7* | 0.0001*** |

**Table (A5): Correlation table between the most abundant genera in the sputum microbiome**

| **Taxon1** | **Taxon2** | **Correlation** | **P-value** |  |
| --- | --- | --- | --- | --- |
| *Fusobacterium* | *Defluviitaleaceae_UC* | 0.7*** | 0.0001*** |  |
| *Fusobacterium* | *Candidate_division* | 0.6 | 0.0001 |  |
| *Fusobacterium* | *Filifactor* | 0.6 | 0.0001 |  |
| *Fusobacterium* | *Haemophilus* | 0.7** | 0.0001*** |  |
| *Fusobacterium* | *Ruminococcaceae* | 0.7** | 0.0001*** |  |
| *Fusobacterium* | *Streptococcus* | 0.3 | 0.03* |  |
| *Moraxella* | *Atopobium* | 0.7** | 0.001** |  |
| *Moraxella* | *Cellulosilyticum* | 0.4 | 0.02* |  |
| *Moraxella* | *Candidate_division* | 0.6 | 0.0001 |  |
| *Moraxella* | *Filifactor* | 0.6 | 0.0001 |  |
| *Moraxella* | *Fretibacterium* | 0.7** | 0.0001** |  |
| *Moraxella* | *Gemella* | 0.6* | 0.0001 |  |
| *Moraxella* | *Lachnoanaerobaculum* | 0.7** | 0.004** |  |
| *Haemophilus* | *Atopobium* | 0.4 | 0.02 | |
| *Haemophilus* | *Leptotrichia* | 0.3 | 0.02 | |
| *Haemophilus* | *Ruminococcaceae* | 0.5 | 0.003 | |
| *Haemophilus* | *Streptococcus* | 0.8* | 0.003** | |
| *Haemophilus* | *Streptomyces* | 0.3 | 0.04 | |
| *Leptotrichia* | *Paracoccus* | 0.7** | 0.001** | |
| *Leptotrichia* | *Streptococcus* | 0.4 | 0.01 | |
| *Leptotrichia* | *Porphyromonas* | 0.6* | 0.0001*** | |
| *Leptotrichia* | *Sphingomonas* | 0.6* | 0.0001*** | |
| *Leptotrichia* | *Catonella* | 0.6* | 0.0001*** | |
| *Leptotrichia* | *Lachnoanaerobaculum* | 0.6* | 0.0001*** | |
| *Leptotrichia* | *Atopobium* | 0.7** | 0.001** | |
| *Leptotrichia* | *Filifactor* | 0.6* | 0.0001*** | |
| *Streptococcus* | *Streptomyces* | 0.4 | 0.01* | |
| *Streptococcus* | *Hungatella* | 0.6* | 0.0001 | |
| *Streptomyces* | *Atopobium* | 0.4 | 0.006 | |
| *Streptomyces* | *Catonella* | 0.6* | 0.0001 | |

##### **Table A6: Samples metadata**

| **Age** | **Diagnosis** | | | **Treatment** | | |
| --- | --- | --- | --- | --- | --- | --- |
|  | **AE-COPD** | **S-COPD** | **P-Value** | **ICS** | **ICS+ABS*** | **P-Value** |
| 40-60 years | 4 | 10 | 0.480 | 10 | 4 | 0.586 |
| >60 years | 13 | 20 |  | 26 | 7 |  |
| AE-COPD: Exacerbated chronic obstructive pulmonary disease group; S-COPD: Stable chronic obstructive pulmonary disease group  AB-ICS: antibiotic with inhaled corticosteroid group; ICS: inhaled corticosteroid group; (*) these specimens were excluded from the study. All subjects were non- smokers for at least 6 months before sampling.  P-value was calculated using the chi-square test | | | | | | |

**Table A7: Sequence analysis for the generated reads and the remaining reads after preprocessing, filtration, denoised, and chimeras removal**.

| **Sample**  **-ID** | **Input** | | **Filtered** | | **Percentage e of input passed**  **filter** | | **Denoised** | | **Merged** | | **Percentage of input merged** | | **Non- chimeric** | | | **Percentage of input non-**  **chimeric** |  |
| --- | --- | --- | --- | --- | --- | --- | --- | --- | --- | --- | --- | --- | --- | --- | --- | --- | --- |
| 86 | 67237 | | 8920 | | 13.27% | | 8379 | | 5039 | | 7.49 | | 4570 | | | 6.8% |  |
| 87 | 18095 | | 6419 | | 35.47% | | 5580 | | 1474 | | 8.15 | | 1036 | | | 5.73% |  |
| 88 | 25247 | | 4970 | | 19.69% | | 4487 | | 2412 | | 9.55 | | 2162 | | | 8.56% |  |
| 89 | 82267 | | 72332 | | 87.92% | | 70556 | | 11390 | | 13.85 | | 8383 | | | 10.19% |  |
| 90 | 86781 | | 78227 | | 90.14% | | 74888 | | 15374 | | 17.72 | | 8213 | | | 9.46% |  |
| 91 | 55781 | | 43554 | | 78.08% | | 42216 | | 23999 | | 43.02 | | 17367 | | | 31.13% |  |
| 92 | 54856 | | 49755 | | 90.7% | | 48272 | | 9158 | | 16.69 | | 6653 | | | 12.13% |  |
| 93 | 31925 | | 28472 | | 89.18% | | 26017 | | 5914 | | 18.52 | | 2043 | | | 6.4% |  |
| 95 | 18557 | | 17071 | | 91.99% | | 16964 | | 61 | | 0.33 | | 55 | | | 0.3% |  |
| 97 | 47099 | | 40705 | | 86.42% | | 39560 | | 23570 | | 50.04 | | 17038 | | | 36.17% |  |
| 99 | 59344 | | 53620 | | 90.35% | | 52285 | | 8732 | | 14.71 | | 7277 | | | 12.26% |  |
| 100 | 36520 | | 29246 | | 80.08% | | 26667 | | 6030 | | 16.51 | | 2133 | | | 5.84% |  |
| 101 | 32057 | | 22716 | | 70.86% | | 21971 | | 1434 | | 4.47 | | 1015 | | | 3.17% |  |
| 102 | 22923 | | 18712 | | 81.63% | | 18117 | | 2622 | | 11.44 | | 1314 | | | 5.73% |  |
| 103 | 63113 | | 56841 | | 90.06% | | 54578 | | 13776 | | 21.83 | | 6466 | | | 10.25% |  |
| 104 | 43794 | | 39509 | | 90.22% | | 38891 | | 715 | | 1.63 | | 378 | | | 0.86% |  |
| 125 | 41990 | | 3950 | | 9.41% | | 3656 | | 2438 | | 5.81 | | 2341 | | | 5.58% |  |
| 126 | 55781 | | 43554 | | 78.08% | | 42216 | | 23999 | | 43.02 | | 17367 | | | 31.13% |  |
| 127 | 13500 | | 5645 | | 41.81% | | 4933 | | 1376 | | 10.19 | | 963 | | | 7.13% |  |
| 128 | 4595 | | 774 | | 16.84% | | 552 | | 85 | | 1.85 | | 85 | | | 1.85% |  |
| 129 | 32057 | | 22716 | | 70.86% | | 21971 | | 1434 | | 4.47 | | 1015 | | | 3.17% |  |
| 130 | 8682 | | 2849 | | 32.82% | | 2526 | | 362 | | 4.17 | | 343 | | | 3.95% |  |
| 131 | 63939 | | 54394 | | 85.07% | | 52904 | | 8822 | | 13.8 | | 7348 | | | 11.49% |  |
| 132 | 72637 | | 8920 | | 13.27% | | 8379 | | 5039 | | 7.49 | | 4563 | | | 6.2% |  |
| 133 | 22165 | | 6719 | | 30.47% | | 5510 | | 1674 | | 7.55 | | 1634 | | | 7.37% |  |
| 134 | 82237 | | 72432 | | 88.07% | | 70456 | | 11931 | | 14.51 | | 8338 | | | 10.14% |  |
| 136 | 64846 | | 59755 | | 92.14% | | 43172 | | 8851 | | 13.64 | | 6356 | | | 9.8% |  |
| 137 | 29415 | | 27071 | | 92.03% | | 12954 | | 75 | | 0.25 | | 58 | | | 0.2% |  |
| 139 | 52079 | | 40715 | | 78.18% | | 33570 | | 23570 | | 45.25 | | 18307 | | | 35.15% |  |
| 141 | 46520 | | 39546 | | 85.08% | | 28267 | | 5978 | | 12.85 | | 2331 | | | 5.01% |  |
| 144 | 23923 | | 17712 | | 74.04% | | 14567 | | 3226 | | 13.48 | | 1413 | | | 5.91% |  |
| 145 | 53113 | | 50541 | | 95.16% | | 53868 | | 14112 | | 26.56 | | 6664 | | | 12.55% |  |
| 148 | | 53794 | | 46709 | | 86.82% | | 32231 | | 721 | | 1.34 | | 389 | 0.72% | | |
| 150 | | 61990 | | 4989 | | 8.41% | | 3446 | | 2938 | | 4.74 | | 2134 | 3.44% | | |
| 151 | | 51780 | | 47454 | | 91.65% | | 47346 | | 29993 | | 57.92 | | 16377 | 31.63% | | |
| 152 | | 23503 | | 8573 | | 36.47% | | 5733 | | 1973 | | 8.39 | | 987 | 4.2% | | |
| 155 | | 7382 | | 3948 | | 53.48% | | 2716 | | 326 | | 4.42 | | 365 | 4.94% | | |
| 157 | | 67319 | | 64493 | | 95.80% | | 67404 | | 8228 | | 12.22 | | 7843 | 11.65% | | |
| 206 | | 77377 | | 35167 | | 45.44% | | 5580 | | 3974 | | 5.13 | | 3773 | 4.87% | | |
| 208 | | 67162 | | 57151 | | 85.09% | | 70556 | | 11950 | | 17.79 | | 8763 | 13.05% | | |
| 213 | | 114024 | | 57333 | | 50.28% | | 21971 | | 5792 | | 5.07 | | 3535 | 3.10% | | |
| N2 | | 114327 | | 46280 | | 40.48% | | 4933 | | 9467 | | 8.28 | | 8859 | 7.75% | | |
| N11 | | 124820 | | 46048 | | 36.89% | | 5580 | | 6847 | | 5.48 | | 5674 | 4.55% | | |
| N21 | | 115300 | | 46300 | | 40.15% | | 2526 | | 9757 | | 8.46 | | 4173 | 3.62% | | |
| N26 | | 77527 | | 35267 | | 45.49% | | 5580 | | 5663 | | 7.30 | | 3556 | 4.59% | | |
| N41 | | 91470 | | 28130 | | 30.75% | | 2526 | | 5671 | | 6.19 | | 2915 | 3.19% | | |
| D15 | | 96723 | | 64284 | | 66.46% | | 21971 | | 6622 | | 6.84 | | 4311 | 4.46% | | |


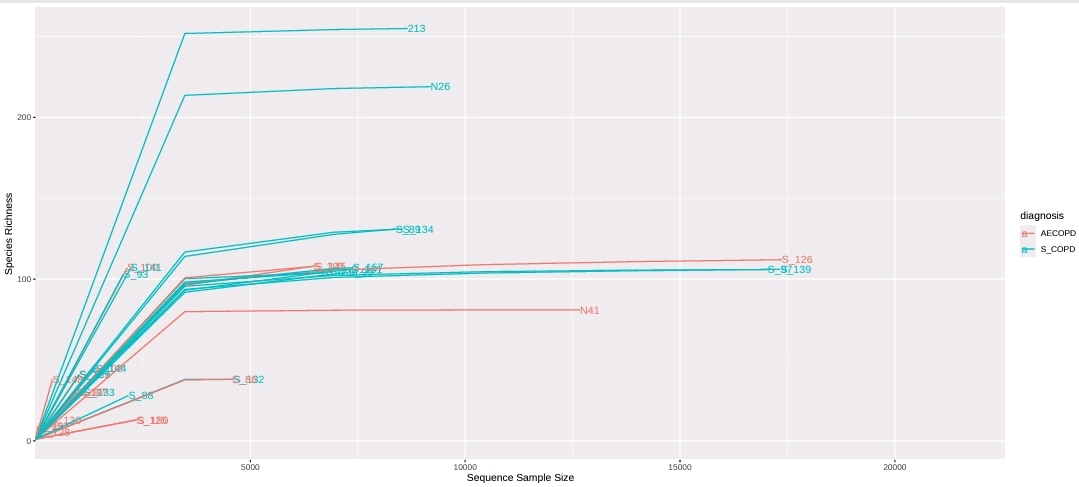


**Figure (S1):** Rarefaction curves of 16S rRNA gene sequences for each sample in both groups (S-COPD, AE-COPD) calculated for ASVs. The vertical axis represents operational taxonomic units, and the horizontal axis represents the sequence of samples score. OTU = operational taxonomic unit. S-COPD group (blue curve) versus the AE-COPD group (red curve).


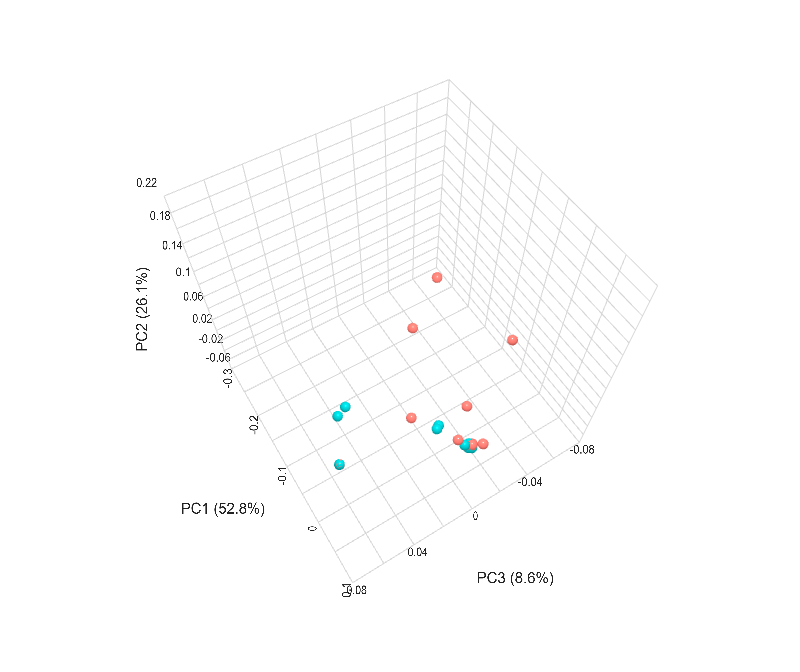


**Figure (S2):** PCoA 3D beta diversity between -COPD and S-COPD based on weighted UniFrac with PREMANOVA as the statistical method, p value = 0.354

**
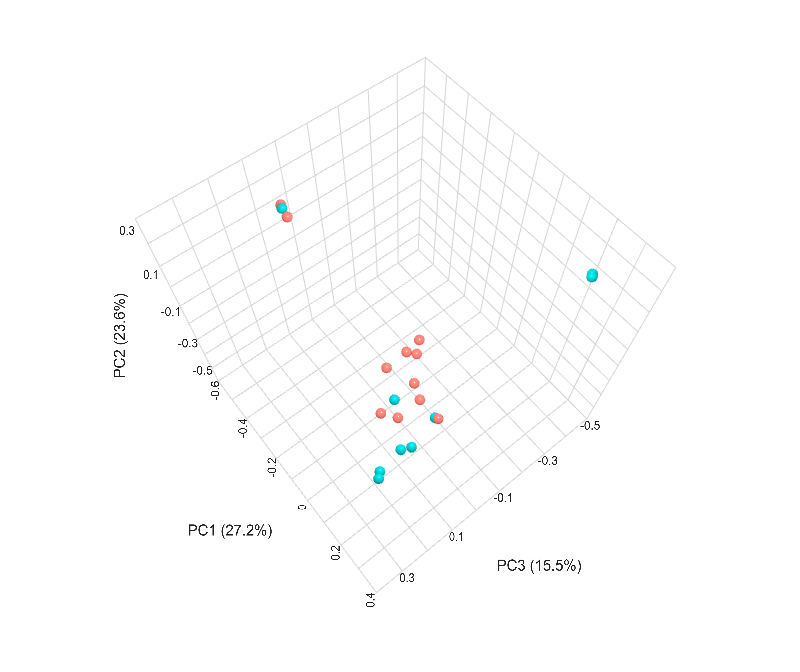
**

**Figure (S3):** PCoA 3D beta diversity between AE-COPD and S-COPD based on unweighted UniFrac with PREMANOVA as the statistical method, p value = 0.009


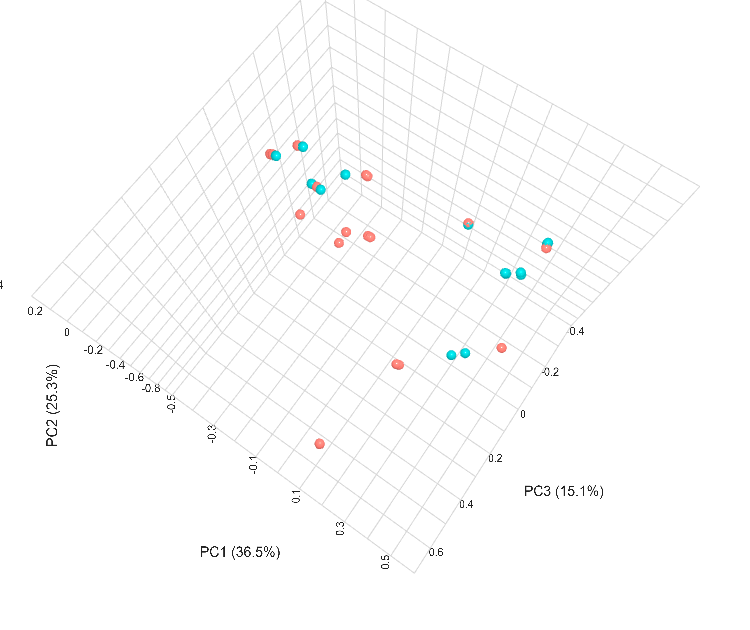


**Figure (S4):** PCoA 3D beta diversity between AE-COPD and S-COPD based on Bray‒Curtis distance

with PREMANOVA as the statistical method, p value = 0.02.


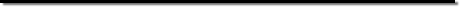


Supplementary Table A8 - The STORMS checklist. An editable version for adaptation and inclusion in publications is available from https://stormsmicrobiome.org

| Number | Item | Recommendation | Item Source | Additional Guidance | Yes/No/NA | Comments or location in manuscript |
| --- | --- | --- | --- | --- | --- | --- |
| **Abstract** | | | | | | |
| 1.0 | Structured or Unstructured Abstract | Abstract should include information on background, methods, results, and conclusions in structured or unstructured format. | STORMS |  | yes | Structured abstract present in the beginning of the manuscript |
| 1.1 | Study Design | State study design in abstract. | STORMS | See 3.0 for additional information on study design. | yes | Abstract and Methods section |
| 1.2 | Sequencing methods | State the strategy used for metagenomic classification. | STORMS | For example, targeted 16S by qPCR or sequencing, shotgun metagenomics, metatranscriptomics, etc. | Yes | The sputum microbiome was analyzed via 16S rRNA gene sequencing |
| 1.3 | Specimens | Describe body site(s) studied. | STORMS |  | yes | Sputum |
| **Introduction** | | | | | | |
| 2.0 | Background and Rationale | Summarize the underlying background, scientific evidence, or theory driving the current hypothesis as well as the study objectives. | STORMS |  | yes | Introduction section |
| 2.1 | Hypotheses | State the pre-specified hypothesis. If the study is exploratory, state any pre-specified study objectives. | STORMS |  | yes | To determine and compare the respiratory microbiome composition in different COPD clinical states by using 16S rRNA gene sequencing and Bioinformatic analysis |
| **Methods** | | | | | | |
| 3.0 | Study Design | Describe the study design. | STORMS | Observational prospective study | Yes | Check methods study design title |
| 3.1 | Participants | State what the population of interest is, and the method by which participants are sampled from that population. Include relevant information on physiological state of the subjects or stage in the life history of disease under study when participants were sampled. | STORMS | The participants in the study, adult male Patients with confirmed diagnosis of COPD, came to the hospital outpatient clinics for routine follow-up visits, scheduled appointments, administration of regular treatments, having respiratory symptoms or for other medical conditions. The participants underwent examinations and were classified based on their clinical presentation in accordance with GOLD criteria (10). Stable COPD (S-COPD) included patients with no acute worsening of respiratory symptoms, evaluated in the outpatient setting for routine follow-up or other non-exacerbation-related reasons. Acute exacerbation COPD (AE-COPD) was defined as an acute worsening of respiratory symptoms beyond normal day-to-day variation, requiring a change in treatment and/or hospital admission.  Sociodemographic and clinical information, including smoking history and the existence of coexisting diseases, respiratory symptoms, exacerbation frequency and therapies, were recorded. The exclusion criteria include patients who received antibiotic therapy (for at least three months without the use of antibiotics for any other reason), immunosuppressive drugs or microbial preparations such as probiotics or prebiotics.; female patients; smokers; patients with history or clinical diagnosis of acute or chronic respiratory diseases, including bronchiectasis, asthma, cystic fibrosis, diffuse bronchiolitis, pulmonary tuberculosis, pulmonary embolism, or pulmonary edema; presented with concurrent pneumonia based on radiographic findings or had a body temperature exceeding 38.0°C at admission; had an active malignancy; suffered from chronic, clinically significant cardiovascular, diabetic, hepatic, renal, or gastrointestinal disorders and had confirmed or suspected immunosuppression or immunodeficiency, whether primary or acquired, including HIV infection . | Yes | Check the updated patients sections |
| 3.2 | Geographic location | State the geographic region(s) where participants were sampled from. | MIxS: geographic location (country and/or sea,region) | Helwan Industrial region, Egypt | yes | Mentioned in Cairo Egypt with the name of the hospital |
| 3.3 | Relevant Dates | State the start and end dates for recruitment, follow-up, and data collection. | STORMS | The study took place from June 2019 to January 2020. Seventy-four patients were included in the study while only 35 patients were analyzed due to, all specimens from Hospital A were excluded, and bad reads from the remaining samples. | yes | Mentioned in study design |
| 3.4 | Eligibility criteria | List any criteria for inclusion and exclusion of recruited participants. | Modified STROBE | The exclusion criteria include patients who received antibiotic therapy (for at least three months without the use of antibiotics for any other reason), immunosuppressive drugs or microbial preparations such as probiotics or prebiotics.; female patients; smokers; patients with history or clinical diagnosis of acute or chronic respiratory diseases, including bronchiectasis, asthma, cystic fibrosis, diffuse bronchiolitis, pulmonary tuberculosis, pulmonary embolism, pulmonary edema, pneumonia based on radiographic findings or had a body temperature exceeding 38.0°C at admission; had an active malignancy; sufferring from chronic, clinically significant cardiovascular, diabetic, hepatic, renal, or gastrointestinal disorders and had confirmed or suspected immunosuppression or immunodeficiency, whether primary or acquired, including HIV infection . | Yes | Check the patients section |
| 3.5 | Antibiotics Usage | List what is known about antibiotics usage before or during sample collection. | STORMS | If participants were excluded due to current or recent antibiotics usage, state this here.  Other factors (e.g. proton pump inhibitors, probiotics, etc.) that may influence the microbiome should also be described as well. | Yes | Check the patients section |
| 3.6 | Analytic sample size | Explain how the final analytic sample size was calculated, including the number of cases and controls if relevant, and reasons for dropout at each stage of the study. This should include the number of individuals in whom microbiome sequencing was attempted and the number in whom microbiome sequencing was successful. | STORMS | Consider use of a flow diagram (see template at https://stormsmicrobiome.org/figures). Also state sample size in abstract.  If power analysis was used to calculate sample size, describe those calculations. | Yes | It is mentioned now that 74 samples were included but only 35 were analyzed due to bad reads |
| 3.7 | Longitudinal Studies | For longitudinal studies, state how many follow-ups were conducted, describe sample size at follow-up by group or condition, and discuss any loss to follow-up. | STORMS | If there is loss to follow-up, discuss the likelihood that drop-out is associated with exposures, treatments, or outcomes of interest. | NA | We didn’t do any follow up. |
| 3.8 | Matching | For matched studies, give matching criteria. | Modified STROBE | "Matched" refers to matching between comparable study participants as cases and controls or exposed / unexposed.  Indicate whether participants were individual or frequency matched and in what ratio were they matched (e.g. 1 case to 1 control). | NA |  |
| 3.9 | Ethics | State the name of the institutional review board that approved the study and protocols, protocol number and date of approval, and procedures for obtaining informed consent from participants. | STORMS |  | Yes | In the methdology under ethical statement |
| 4.0 | Laboratory methods | State the laboratory/center where laboratory work was done. | STORMS | Provide a reference to complete lab protocols if previously published elsewhere such as on protocols.io. Note any modifications of lab protocols and the reason for protocol modifications. | Yes | References for all the methods used are already written in the manuscript. |
| 4.1 | Specimen collection | State the body site(s) sampled from and how specimens were collected. | MIxS: sample collection device or method; host body site | induced sputum samples were collected (11). A total of 35 samples were assigned to two clinical states: S-COPD (n = 17) and AE-COPD (n = 18). The samples were taken early on the first day. Aliquots (0.5 ml) of sputum samples were taken in sterile sample bottles and stored at − 80 °C for DNA extraction. | Yes | Sputum |
| 4.2 | Shipping | Describe how samples were stored and shipped to the laboratory. | STORMS | Sputum samples were subjected to DNA extraction and PCR amplification before being shipped to the IGA Technology Services Company (Udine, Italy). Extracted DNA were sealed in cryo-tubes in ice packs. It took about two weeks to reach IGA Technology Services | Yes | in sterile sample bottles and were transferred in in ice packs |
| 4.3 | Storage | Describe how the laboratory stored samples, including time between collection and storage and any preservation buffers or refrigeration used. | STORMS | State where each procedure or lot of samples was done if not all in the same place.  Include reagent/lot/catalogue #s for storage buffers. | Yes | sputum samples were taken and stored at − 80 °C for DNA extraction, and the remaining samples were subjected to routine culture |
| 4.4 | DNA extraction | Provide DNA extraction method, including kit and version if relevant. | MIxS: nucleic acid extraction | If any DNA quantification methods were used prior to DNA amplification or at the pooling step of library preparation, state so here. | Yes | Mentioned in methods (2.4) The quality and quantity of the extracted DNA were evaluated via a NanoDrop system (NanoDrop Technology, USA). It was then visualized via 2% agarose gel electrophoresis |
| 4.5 | Human DNA sequence depletion or microbial DNA enrichment | Describe whether human DNA sequence depletion or enrichment of microbial or viral DNA was performed. | STORMS |  | Yes | enrichment |
| 4.6 | Primer selection | Provide primer selection and DNA amplification methods as well as variable region sequenced (if applicable). | MIxS: pcr primers |  | Yes | In PCR section methods |
| 4.7 | Positive Controls | Describe any positive controls (mock communities) if used. | STORMS | If used, should be deposited under guidance provided in the 8.X items. | Yes | Controls included the ZymoBIOMICS TM D6311 Microbial DNA Community Standard II (mock community) |
| 4.8 | Negative Controls | Describe any negative controls if used. | STORMS | If used, should be deposited under guidance provided in the 8.X items. | Yes | A no-template negative control, which was included in each PCR plate. |
| 4.9 | Contaminant mitigation and identification | Provide any laboratory or computational methods used to control for or identify microbiome contamination from the environment, reagents, or laboratory. | STORMS | Includes filtering of reagents and other steps to minimize contamination. It is relevant to state whether the specimens of interest have low microbial load, which makes contamination especially relevant. | Yes | Mentioned in methods |
| 4.10 | Replication | Describe any biological or technical replicates included in the sequencing, including which steps were replicated between them. | STORMS | Replication may be biological (redundant biological specimens) or technical (aliquots taken at different stages of analysis) and used in extraction, sequencing, preprocessing, and/or data analysis. | No |  |
| 4.11 | Sequencing strategy | Major divisions of strategy, such as shotgun or amplicon sequencing. | MIxS: sequencing method | For amplicon sequencing (for example, 16S variable region), state the region selected. State the model of sequencer used. | yes | Amplicon sequencing method by Illumina MiSeq (mentioned in sequencing methods) |
| 4.12 | Sequencing methods | State whether experimental quantification was used (QMP/cell count based, spike-in based) or whether relative abundance methods were applied. | STORMS | These include read length, sequencing depth per sample (average and minimum), whether reads are paired, and other parameters. | yes | Using paired-end Illumina MiSeq sequencing on an Illumina MiSeq instrument (Illumina Inc., San Diego, CA, USA), the 16S rRNA was sequenced. All parameters are mentioned in supplementary table (A7 and figure S1) |
| 4.13 | Batch effects | Detail any blocking or randomization used in study design to avoid confounding of batches with exposures or outcomes. Discuss any likely sources of batch effects, if known. | STORMS | Sources of batch effects include sample collection, storage, library preparation, and sequencing and are commonly unavoidable in all but the smallest of studies. | NA |  |
| 4.14 | Metatranscriptomics | Detail whether any mRNA enrichment was performed and whether/how retrotranscription was performed prior to sequencing. Provide size range of isolated transcripts. Describe whether the sequencing library was stranded or not. Provide details on sequencing methods and platforms. | STORMS | Provide details on any internal standards which may have been used as well as parameters and versions of any software or databases used. | NA |  |
| 4.15 | Metaproteomics | Detail which protease was used for digestion. Provide details on proteomic methods and platforms (e.g. LC-MS/MS, instrument type, column type, mass range, resolution, scan speed, maximum injection time, isolation window, normalised collision energy, and resolution). | STORMS | Provide details on any internal standards which may have been used as well as parameters and versions of any software or databases used. | NA |  |
| 4.16 | Metabolomics | Specify the analytic method used (such as nuclear magnetic resonance spectroscopy or mass spectrometry). For mass spectrometry, detail which fractions were obtained (polar and/or non-polar) and how these were analyzed. Provide details on metabolomics methods and platforms (e.g. derivatization, instrument type, injection type, column type and instrument settings). | STORMS | Provide details on any internal standards which may have been used as well as parameters and versions of any software or databases used. | NA |  |
| 5.0 | Data sources/  measurement | For each non-microbiome variable, including the health condition, intervention, or other variable of interest, state how it was defined, how it was measured or collected, and any transformations applied to the variable prior to analysis. | MIxS: host disease status | State any sources of potential bias in measurements, for example multiple interviewers or measurement instruments, and whether these potential biases were assessed or accounted for in study design.  Use terms from a standardized ontology such as the Experimental Factor Ontology (https://www.ebi.ac.uk/efo/) to describe variables of interest in a standardized format. | No |  |
| 6.0 | Research design for causal inference | Discuss any potential for confounding by variables that may influence both the outcome and exposure of interest. State any variables controlled for and the rationale for controlling for them. | STORMS | For causal inference, this item refers to describing the assumptions that would be required to draw causal inferences from observational data. See Vujkovic-Cvijin, I., Sklar, J., Jiang, L. et al. Host variables confound gut microbiota studies of human disease. Nature 587, 448–454 (2020). https://doi.org/10.1038/s41586-020-2881-9 for more details on confounding in observational microbiome studies.  For example, hypothesized confounders may be controlled for by multivariable adjustment. Consider using a directed acyclic graph (DAG) to describe your causal model and justify any variables controlled for. DAGs can be made using www.dagitty.net. | NA |  |
| 6.1 | Selection bias | Discuss potential for selection or survival bias. | STORMS | Selection bias can occur when some members of the target study population are more likely to be included in the study/final analytic sample than others. Some examples include survival bias (where part of the target study population is more likely to die before they can be studied), convenience sampling (where members of the target study population are not selected at random), and loss to follow-up (when probability of dropping out is related to one of the things being studied). | NA |  |
| 7.0 | Bioinformatic and Statistical Methods | Describe any transformations to quantitative variables used in analyses (e.g. use of percentages instead of counts, normalization, rarefaction, categorization). | STORMS | If a variable is analyzed using different transformations, state rationale for the transformation and for each analyses which version of the variable is used.  In case of any complex or multistep transformations, give enumerated instructions for reproducing those transformations. | Yes | The rarefaction curves confirmed that the sequenced samples covered the dominant members of the bacterial communities (Fig. 1 supplementary). |
| 7.1 | Quality Control | Describe any methods to identify or filter low quality reads or samples. | MIxS: sequence quality check | If samples were excluded based on quality or read depth, list the criteria used, the number of samples excluded, and the final sample size after quality control. | Yes | (2.6) sequence processing and quality filtering were performed via the “Quantitative Insights into Microbial Ecology” (QIIME2R version 0.99.21) pipeline to extract taxonomic information. The "join paired ends.py" argument was used to fuse overlapping paired-end 16S rRNA gene sequences. Sequences with ambiguous reads (N), low-quality sequences end with mismatched forward or reverse primers, failed sequence reads, barcodes, and primers were eliminated for quality control. Sequences under 200 bp were also trimmed via the "QIIME script split_libraries.py calls" parameter (quality score < 25). Seventy four participants were included in the study, but only 35 were analyzed (section 2.1) |
| 7.2 | Sequence analysis | Describe any taxonomic, functional profiling, or other sequence analysis performed. | MIxS: feature prediction; similarity search method |  | yes | Find (2.7) statistical analysis |
| 7.3 | Statistical methods | Describe all statistical methods. | Modified STROBE | Describe any statistical tests used, exploratory data analysis performed, dimension reduction methods/unsupervised analysis, alpha/beta metrics, and/or methods for adjusting for measurement bias.  If multiple statistical methods are possible, discuss why the methods used were selected.  If a multiple hypothesis testing correction method was used, describe the type of correction used.  State which taxonomic levels are analyzed. | Yes | Mentioned under the title statistical analysis |
| 7.4 | Longitudinal analysis | If the study is longitudinal, include a section that explicitly states what analysis methods were used (if any) to account for grouping of measurements by individual or patterns over time. | STORMS |  | NA |  |
| 7.5 | Subgroup analysis | Describe any methods used to examine subgroups and interactions. | STROBE |  | NA |  |
| 7.6 | Missing data | Explain how missing data were addressed. | STROBE | "Missing data" refers to participant measurements such as covariates, exposures, outcomes, or time points that should have been collected but were not, not to zeros in taxonomic abundance tables or data points not applicable to that observation. | NA |  |
| 7.7 | Sensitivity analyses | Describe any sensitivity analyses. | STROBE |  | NA |  |
| 7.8 | Findings | State criteria used to select findings for reporting. | STORMS | For example, false discovery rate with total number of tests, effect size threshold, significance threshold, microbes of interest. | Yes | (2.7) To control the false-discovery rate across all tested ASVs, p-values were converted to FDR-values. An FDR threshold of q < 0.05 was considered statistically significant unless stated otherwise. |
| 7.9 | Software | Cite all software (including read mapping software) and databases (including any used for taxonomic reference or annotating amplicons, if applicable) used. Include version numbers. | Modified STREGA | Installed packages, add-ons or libraries should be stated and cited in addition to the software used.  All parameters employed that differ from the default of that software/version should be provided.  This is in addition to, not a replacement for, publishing of code as outlined in the section Reproducible Research. | Yes | statistical analyses were performed with https://www.microbiomeanalyst.ca. The Kruskal‒Wallis (KW) test and the nonparametric Mann‒Whitney test were used to identify species with significant differences between two or more groups, respectively. Diversity and differential-abundance analyses used unrarefied ASV table with normalization (R package). “Quantitative Insights into Microbial Ecology” (QIIME2R version 0.99.21) (15) pipeline to extract taxonomic information. The "join paired ends.py" argument was used to fuse overlapping paired-end 16S rRNA gene sequences. Sequences were grouped into Amplicon Sequence Variant (ASV) clusters and aligned via the SILVA alignment database (http://www.arb-silva.de/). The truncation length parameters of DADA2 were p-trunc-len-f 280 and p-trunc-len-r 220. |
| 8.0 | Reproducible research | Make a statement about whether and how others can reproduce the reported analysis. | STORMS | Any protected information that has been excluded or provided under controlled access should be listed along with any relevant data access procedures. "On request from authors" is not sufficiently detailed; formal data access procedures and conditions should be defined.  If data are unavailable, state so clearly.  Consider using a specialized rubric for reproducible research (such as: https://mbio.asm.org/content/9/3/e00525-18.short).  Consider preregistering the study protocol (such as on osf.io or https://plos.org/open-science/preregistration/). | yes | All methods are clearly stated. No information has been excluded. Bioproject the accession number PRJNA1021628 in the NCBI Bioproject (http://www.ncbi.nlm.nih.gov/bioproject). |
| 8.1 | Raw data access | State where raw data may be accessed including demultiplexing information. | STORMS | Robust, long-term databases such as those hosted by NCBI and EBI are preferred. If using a private repository, provide rationale. | yes | The sequence data for the raw data were deposited with the accession number PRJNA1021628 , also some of the raw data are presented in the supplementary material |
| 8.2 | Processed data access | State where processed data may be accessed. | STORMS | Unfiltered data should be provided.  Robust, long-term databases such as those hosted by NCBI and EBI-EMBL are preferred. Repositories like zenodo (https://zenodo.org/) or publisso (https://www.publisso.de/en/working-for-you/doi-service/)  can be used to provide a DOI and long-term storage for processed datasets, even those which cannot be published openly. | yes | accession number PRJNA1021628 |
| 8.3 | Participant data access | State where individual participant data such as demographics and other covariates may be accessed, and how they can be matched to the microbiome data. | STORMS | If re-categorized, transformed, or otherwise derived variables were used in the analysis, these variables or code for deriving them should be provided.  Examples of how participant data can be matched to microbiome data are: using the same set of anonymized identifiers, or using different anonymized identifiers but providing a map.  Provided data should be sufficient to independently replicate the current analysis. | Yes | Check the supplementary material |
| 8.4 | Source code access | State where code may be accessed. | STORMS | If a standard or formalized workflow was employed, reference it here. | NA |  |
| 8.5 | Full results | Provide full results of all analyses, in computer-readable format, in supplementary materials. | STORMS | For example, any fold-changes, p-values, or FDR values calculated, provided as a spreadsheet.  Use a machine-readable, plain-text format such as csv or tsv. | Yes | Full results are stated in results section and in supplementary materials |
| **Results** | | | | | | |
| 9.0 | Descriptive data | Give characteristics of study participants (e.g. dietary, demographic, clinical, social) and information on exposures and potential confounders. | STROBE | Typically reported in a table included in the paper or as a supplementary table. Indicate number of participants with missing data for each variable of interest.  This includes environmental and lifestyle factors that may affect the relationship between the microbiome and the condition of interest. Participant diet and medication use should be summarized, if known.  At minimum, age and sex of all participants should be summarized. | yes | (Under the topic metadata of patients. Supplementary A6) |
| 10.0 | Microbiome data | Report descriptive findings for microbiome analyses with all applicable outcomes and covariates. | STORMS | This includes measures of diversity as well as relative abundances. These descriptive findings should be reported both for the sample overall and for individual groups. | yes | Mentioned clearly in the results section |
| 10.1 | Taxonomy | Identify taxonomy using standardized taxon classifications that are sufficient to uniquely identify taxa. | STORMS | If not using full taxonomic hierarchy, make sure it is clear whether names stated are species, genera, family, etc.  Italicize genus/species pairs. Consult journal guidelines or standardized references on taxonomic nomenclature. For instance, https://wwwnc.cdc.gov/eid/page/scientific-nomenclature | yes | Mentioned clearly in the results section |
| 10.2 | Differential abundance | Report results of differential abundance analysis by the variable of interest and (if applicable) by time, clearly indicating the direction of change and total number of taxa tested. | STORMS | If there are more than two groups, include omnibus (multigroup) test results if applicable to the research question.  If applicable, reported effect sizes should include a measure of uncertainty such as the confidence interval. | Yes | Mentioned clearly in the results section |
| 10.3 | Other data types | Report other data analyzed–e.g. metabolic function, functional potential, MAG assembly, and RNAseq. | STORMS |  | NA |  |
| 10.4 | Other statistical analysis | Report any statistical data analysis not covered above. | STORMS | This could include subgroup analysis, sensitivity analyses, and cluster analysis.  Visualizations should be easily interpretable and colorblind-friendly. The caption and/or main text should provide a detailed description of visualizations for visually-impaired readers. | NA |  |
| **Discussion** | | | | | | |
| 11.0 | Key results | Summarise key results with reference to study objectives | STROBE |  | yes | Stated clearly in the discussion section |
| 12.0 | Interpretation | Give a cautious overall interpretation of results considering objectives, limitations, multiplicity of analyses, results from similar studies, and other relevant evidence. | STROBE | Define or clarify any subjective terms such as "dominant," "dysbiosis," and similar words used in interpretation of results.  When interpreting the findings, consider how the interpretation of the findings may be summarized or quoted for the general public such as in press releases or news articles.  If causal language is used in the interpretation (such as "alters," "affects," "results in," "causes," or "impacts"), assumptions made for causal inference should be explicitly stated as part of 6.0 and 13.0.  Distinguish between function potential (ie inferred from metagenomics) and observed activity (ie metatranscriptomic, metabolomic, proteomic) if discussing microbial function. | yes | Mentioned clearly in discussion. |
| 13.0 | Limitations | Discuss limitations of the study, taking into account sources of potential bias or imprecision. | STROBE | Also consider limitations resulting from the methods (especially novel methods), the study design, and the sample size. | yes | Mentioned clearly in last paragraph of discussion |
| 13.1 | Bias | Discuss any potential for bias to influence study findings. | STORMS | May include sampling method, representativeness of study participants, or potential confounding. | NA |  |
| 13.2 | Generalizability | Discuss the generalisability (external validity) of the study results | STROBE | To what populations or other settings do you expect the conclusions to generalize? |  |  |
| 14.0 | Ongoing/future work | Describe potential future research or ongoing research based on the study's findings. | STORMS |  | Yes | Check conclusion |
| **Other information** | | | | | | |
| 15.0 | Funding | Give the source of funding and the role of the funders for the present study and, if applicable, for the original study on which the present article is based | STROBE |  | No |  |
| 15.1 | Acknowledgements | Include acknowledgements of those who contributed to the research but did not meet critera for authorship. | STORMS | For general guidelines on authorship, see http://www.icmje.org and https://www.elsevier.com/authors/journal-authors/policies-and-ethics/credit-author-statement | yes | We acknowledge the support of medical staff and doctors in the two hospitals for providing diagnosis and sputum samples. |
| 15.2 | Conflicts of Interest | Include a conflicts of interest statement. | STORMS |  | yes | I declare that the authors have no competing interests as defined by Springer (Not Applicable), or other interests that might be perceived to influence the results and/or discussion reported in this paper. |
| 16.0 | Supplements | Indicate where supplements may be accessed and what materials they contain. | STORMS |  | yes | Supplements may be accessed at the end of manuscript. It includes: tables (A1-A7) and Figures (S1-S4). In addition supplementary methods |
| 17.0 | Supplementary data | Provide supplementary data files of results with for all taxa and all outcome variables analyzed. Indicate the taxonomic level of all taxa. | STORMS | Depending on the analysis performed, examples of the supplemental results included could be mean relative abundance, differential abundance, raw p-value, multiple hypothesis testing-adjusted p-values, and standard error.  All discussed taxa should include the taxonomic level (e.g. class, order, genus). | yes | Check Supplemental tables (A1-A5) |
